# Quality indicators in surgical oncology: systematic review of measures used to compare quality across hospitals

**DOI:** 10.1093/bjsopen/zrae009

**Published:** 2024-03-21

**Authors:** Megan McLeod, Kari Leung, C S Pramesh, Peter Kingham, Miriam Mutebi, Julie Torode, Andre Ilbawi, Jade Chakowa, Richard Sullivan, Ajay Aggarwal

**Affiliations:** Department of Health Policy, London School of Economics and Political Science, London, UK; Department of Otolaryngology—Head & Neck Surgery, Vanderbilt University Medical Center, Nashville, Tennessee, USA; Department of Oncology, Guy’s & St Thomas’ NHS Trust, London, UK; Tata Memorial Centre, Homi Bhabha National Institute, Mumbai, India; Department of Surgery, Memorial Sloan Kettering Cancer Center, New York, New York, USA; Department of Surgery, Aga Khan University, Nairobi, Kenya; Institute of Cancer Policy, Centre for Cancer, Society & Public Health, King’s College London, London, UK; Department of Universal Health Coverage, World Health Organization, Geneva, Switzerland; City Cancer Challenge, Geneva, Switzerland; Institute of Cancer Policy, Global Oncology Group, Centre for Cancer, Society & Public Health, King’s College London, London, UK; Department of Health Services Research and Policy, London School of Hygiene and Tropical Medicine, London, UK

## Abstract

**Background:**

Measurement and reporting of quality indicators at the hospital level has been shown to improve outcomes and support patient choice. Although there are many studies validating individual quality indicators, there has been no systematic approach to understanding what quality indicators exist for surgical oncology and no standardization for their use. The aim of this study was to review quality indicators used to assess variation in quality in surgical oncology care across hospitals or regions. It also sought to describe the aims of these studies and what, if any, feedback was offered to the analysed groups.

**Methods:**

A literature search was performed to identify studies published between 1 January 2000 and 23 October 2023 that applied surgical quality indicators to detect variation in cancer care at the hospital or regional level.

**Results:**

A total of 89 studies assessed 91 unique quality indicators that fell into the following Donabedian domains: process indicators (58; 64%); outcome indicators (26; 29%); structure indicators (6; 7%); and structure and outcome indicators (1; 1%). Purposes of evaluating variation included: identifying outliers (43; 48%); comparing centres with a benchmark (14; 16%); and supplying evidence of practice variation (29; 33%). Only 23 studies (26%) reported providing the results of their analyses back to those supplying data.

**Conclusion:**

Comparisons of quality in surgical oncology within and among hospitals and regions have been undertaken in high-income countries. Quality indicators tended to be process measures and reporting focused on identifying outlying hospitals. Few studies offered feedback to data suppliers.

## Introduction

The Institute of Medicine (IoM) posits six dimensions of healthcare quality; namely, that it should be safe, timely, effective, efficient, equitable, and patient-centred (STEEEP)^1^. It is estimated that over 9 million cancer cases worldwide had an indication for surgery in 2018 and that number is projected to increase to more than 13 million by 2040^2^. In cancer care, low-quality treatment leads to poor survival rates, higher rates of morbidity, and higher costs both to the individual and society^3,4^. Cancer care typically requires STEEEP coordination of several modalities of treatment (that is surgery, chemotherapy, and radiation therapy) to treat the cancer, prevent recurrence, and avoid toxicities. The growing burden of cancer, the complexity of treatment pathways, and the need for multidisciplinary coordination means that developing indicators that adequately measure all aspects of high-quality oncological care delivery is essential.

Donabedian framed measures of quality according to whether they assess the maintenance of the organizational and physical structure necessary for the provision of quality care, the execution of processes that are known to contribute to high-quality care, or the achievement of outcomes consistent with provision of quality care^5^. These categories are typically referred to as structure, process, and outcome measures respectively. Some models have gone further, leading to 12 domains of quality to appraise cancer care^6^.

With the exponential growth in data collected via electronic medical records, computerized billing, and online disease-specific registries has come an opportunity to measure, report, and compare quality on both a larger and more granular scale than ever before. In the USA, the American College of Surgeons maintains the National Surgical Quality Improvement Program (NSQIP)^7^. The Netherlands and the UK conduct quality audits of specific diseases, especially cancers^8,9^.

While most academic studies in the field of quality measurement and improvement have focused on developing and validating quality indicators, analyses have begun to focus on comparing quality across organizations or regions of care provision^10^. However, no study has yet explored which quality indicators are being used to assess quality in surgical oncology, which measures meaningfully detect variation between organizations or regions, and what, if anything, comes from identification of this variation^11^.

This aim of this study was to conduct a review of the quality indicators being routinely used to assess variation in quality in surgical oncology care across hospitals or regions with a view to inform standardization of performance measurement in surgical oncology. Second, it sought to identify the sources of data used in these analyses and the entities responsible for producing and maintaining the databases. Lastly, it sought to describe the aims identified in studies seeking to establish hospital or regional variation in quality indicators and what, if any, feedback the authors of these studies offer to the hospitals or regions they analysed to support quality improvement.

## Methods

This review was exempt from Research Ethics Committee review per institutional Research Ethics Policy and Procedures and was approved at the departmental level by the Department of Health Policy.

### Search strategy

This study followed the PRISMA and AMSTAR 2 (‘a measurement tool to assess systematic reviews, version 2’) guidelines^12,13^. A literature search was performed to identify studies published between 1 January 2000 and 31 October 2023 that utilized quality indicators to assess surgical oncology care. The search strategy was created using the population/intervention/comparator/outcomes/study design (PICOS) framework outlined in the Cochrane Handbook (*Table 1*)^14^. The databases searched were Embase via the Ovid interface and MEDLINE via the PubMed interface. The full search strategy is available in *Appendix S1*.

**Table 1:** Outline of population/intervention/comparator/outcomes/study design (‘PICOS’)

**Population**
Hospitals or regions providing cancer care to adult patients (at least 18 years old)
**Intervention**
Comparing quality of surgical oncology care
**Comparator**
Other hospitals, regions, or regional/national/international averages
**Outcomes**
Performance on quality indicators
**Study design**
Cohort studies

### Study selection

Inclusion and exclusion criteria were developed according to the published literature on quality in cancer care in consultation with a clinical oncologist and a surgeon who are experts in the measurement of care quality and experienced in conducting systematic reviews. These criteria are outlined in *Table 2*.

**Table 2:** Inclusion and exclusion criteria

	Inclusion	Exclusion
Date	1 January 2000–23 October 2023	Before 2000
Exposure of interest	Quality assessment of surgical oncology care	Not oncological care Not surgical care (including surgical pathology) Development or validation of quality indicators Associations of quality indicator achievement with patient or surgical approach factors
Geographical location	Hospital or regional	National or international
Language	English language only	Any language other than English
Participants	Adult patients (at least 18 years old)	Paediatric patients
Peer review	Peer-reviewed studies	Non-peer reviewed studies Systematic reviews Grey literature
Reported outcomes	Variation in quality indicators	Comparison of surgical approaches or case-mix or individual surgeon variation
Setting	Clinical/administrative/quality data sets	Single surgeon databases
Study design	Cohort studies	Any study design other than cohort study
Type of publication	Original studies and abstracts	Editorials, letters, news, comments, case reports, commentaries, and narrative reviews Subgroup and post-hoc analyses Systematic reviews/meta-analyses

Original investigations of adult patients (at least 18 years old) that applied surgical quality indicators to detect variation in cancer care at the centre (hospital) or regional level, including those that used quality indicators to benchmark individual centres against a national average, were included.

Studies that only developed or validated quality indicators (and did not compare centres or regions), that only associated achievement of quality indicators with patient or surgical factors (for example co-morbidities, surgical approaches, and preoperative or postoperative interventions), or that solely focused on surgical pathology quality indicators were excluded.

The abstracts and titles of studies retrieved from the literature search were screened for inclusion in full-text review by M.Mc. and K.L. Full-text records were then evaluated against inclusion criteria. Senior reviewers (A.A. and R.S.) were consulted to resolve uncertainties.

### Data extraction

Data were extracted by M.Mc. and K.L. using a predefined and piloted data collection form regarding study characteristics (country, number of hospitals and patients included, tumour type(s), tumour stage, source(s) of data, entity that produced or maintained the database, aims of the study, and method of data visualization), quality indicators and their definitions, characteristics of each quality indicator, and the intended use of the study’s results. Quality indicator definitions were recorded as reported by the investigators of each study. Uncertainties were resolved by consensus meetings with senior reviewers (A.A. and R.S.). Screening and data extraction were conducted using Covidence software.

### Categorization of results

Quality indicators were categorized according to aspect of the care pathway (preoperative, intraoperative, or postoperative), Donabedian domain (structure, process, or outcome), and Quality Indicators in Cancer Care (QICC) domain^5,6^. This was intended to aid in mapping quality indicators to which provider(s) and resources were required for measurement and/or achievement of the quality indicators. QICC domains were defined according to the description of Chiew *et al*.^6^ with one exception. Two domains—safety errors and adverse events (for example surgical morbidity and mortality) and disease-specific outcomes (for example local recurrence rate)—were felt to be too similar in practice to consistently categorize measures. Therefore, these domains were combined.

Quality indicators identified in the literature were also compared with cancer quality indicators endorsed by the National Quality Forum (NQF), the Centers for Medicare and Medicaid Services (CMS), and Healthcare Improvement Scotland (HIS)^15–17^.

The intended use of a study’s results was categorized as either exploratory (for example evaluating the ability of a metric to detect inter-hospital variation) or presenting results of an established quality improvement or reporting programme (for example the results of the Dutch Surgical Colorectal Audit). Whether a study’s authors explicitly commented on providing the results of their study to the studied groups was also noted.

### Risk of bias

A risk-of-bias assessment was not performed for this review as it was intended to provide an overview of the quality indicator landscape in surgical oncology rather than a conclusion by combining studies.

### Statistical analysis

Counts and percentages were calculated for categorical variables. Medians and interquartile ranges were calculated for continuous variables. Continuous variables were also analysed graphically using histograms. All analyses were performed using Stata17.

## Results

**Fig. 1:**
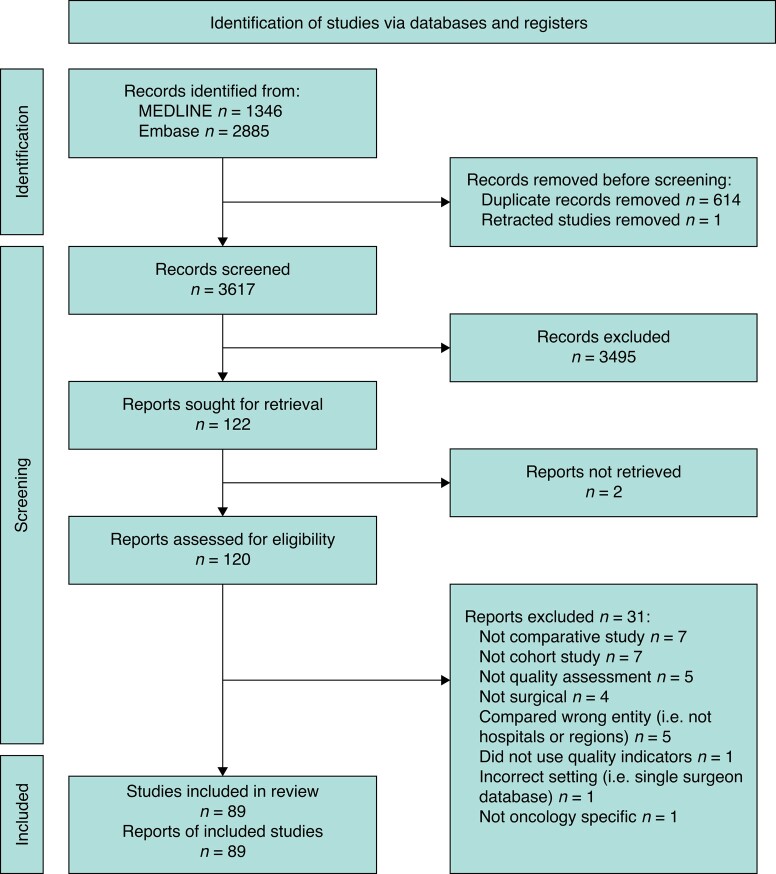
PRISMA flow chart This figure illustrates the process of identifying, screening, and including or excluding studies based upon the defined inclusion and exclusion criteria.

Of the 3617 articles retrieved, 120 full-text articles were reviewed and 89 met the inclusion criteria. *Figure 1* summarizes the literature search and screening process, including the reasons for exclusion during full-text review.

### Study characteristics

Characteristics of the included studies are summarized in *Table 3*. A total of 55 studies (62%) took place in the USA, 15 (17%) in the Netherlands, six (7%) in the UK, and 13 (15%) in other countries.

**Table 3:** Characteristics of included studies

**Country**	
USA	55 (62)
Netherlands	15 (17)
UK	6 (7)
Canada	3 (3)
Belgium	2 (2)
Other	7 (8)
**Level of comparison**	
Between hospital variation	82 (92)
Between geographical region variation	5 (6)
Single hospital to national average	1 (1)
Between provider networks	1 (1)
**Tumour types evaluated**	
Colorectal	26 (29)
Lung	14 (16)
Breast	14 (16)
Multiple	7 (8)
Pancreas	6 (7)
Oesophageal	3 (3)
Head and neck	3 (3)
Prostate	3 (3)
Gynaecological	3 (3)
Gastric	2 (2)
Upper GI tract	2 (2)
Renal	2 (2)
Skin	2 (2)
Other	2 (2)
**Stage of patients evaluated**	
I–IV	46 (58)
I–III	18 (23)
I–II	12 (15)
III–IV	2 (2)
II–IV	1 (1)
**Number of hospitals evaluated, median (i.q.r.)**	148 (28–809)
**Number of regions evaluated, median (i.q.r.)**	5 (5–5)
**Number of patients evaluated, median (i.q.r.)**	12 839 (3035–40 892)

Values are *n* (%) unless otherwise indicated. GI, gastrointestinal; i.q.r., interquartile range.

Most studies reported variation in quality indicators between centres or hospitals (82; 92%), five (6%) reported variation in quality indicators between geographical regions, one (1%) compared a single hospital with the national average, and one (1%) compared provider networks. The median number of hospitals evaluated was 148 (interquartile range 28–809). The median number of patients included was 12 839 (interquartile range 3035–40 892). Most studies (48; 54%) evaluated fewer than 10 000 patients (see *Fig. S1*). Only 14 studies (16%) evaluated more than 100 000 patients^18–30^.

Studies evaluated 16 tumour types in total. Surgery for colorectal tumours was most frequently evaluated (26; 29%)^19–22,30–52^, followed by breast tumours (14; 16%)^53–66^ and lung tumours (14; 16%)^23–26,50–52,67–78^. A total of 46 studies (58%) evaluated patients in all cancer stages, 18 studies (20%) included patients with stage I–III cancer, 12 studies (15%) included only patients with stage I–II cancer, and two studies (2%) only included patients with stage III or IV cancer (ovarian)^29^.

The data used to compare centres or regions primarily came from national (64; 72%) or regional (16; 18%) clinical data registries (*Table 4*). These registries included the US National Cancer Database (22), various Dutch cancer audits (8), and the US NSQIP (6). A total of ten studies (11%) utilized hospital administrative data, nine studies (10%) used medical records, eight studies (9%) used hospital/insurer billing data, and one study (1%) used survey data^75^.

**Table 4:** Data and database sources

**Data source(s)**	
National clinical data registry	64 (72)
Regional clinical data registry	16 (18)
Hospital administrative data	10 (11)
Medical records	9 (10)
Hospital/insurer billing data	8 (9)
Patient survey	1 (1)
**Entity that produced/maintained database**	
Specialty society	15 (19)
National government	9 (10)
Not-for-profit organization	6 (7)
Quality collaborative (self-organized group of hospitals)	6 (7)
State or regional government	5 (6)
Insurance organization (public or private)	3 (3)
Other single entity	3 (3)
Joint effort	
Specialty society and not-for-profit organization	24 (27)
Quality collaborative and insurance organization	6 (7)
National government and insurance organization	3 (3)
National government and state or regional government	2 (2)
National government, state or regional government, and insurance organization	2 (2)
Other combination	5 (6)

Values are *n* (%).

Data collection and maintenance were most often facilitated through collaboration of a specialty society and a not-for-profit organization (24; 27%). Governments seldom took on this role (national governments, nine (10%); and state/regional governments, five (6%)). Specialty societies included the American College of Surgeons, the Dutch Society of Lung Surgeons, and the German Society of General and Visceral Surgery.

### Characteristics of quality indicators

In total, the 89 included studies assessed 91 unique quality indicators. *Table 5* quantifies the categorization of these quality indicators by Donabedian and cancer care pathway domains. More than half of the quality indicators were process indicators (58; 64%). Process indicators included measures such as percentage of patients achieving negative margins, waiting times for surgery, and adequate number of follow-up visits. Outcome quality indicators were also well represented (26; 29%). Outcome quality indicators included failure-to-rescue rates (that is patient deaths after complications), 30- and 90-day mortality rates, and 5-year overall survival. Few quality indicators were classed as structure indicators (6; 7%) and one (1%) fell into both the structure and outcome domains^48^. Structure indicators included the availability of psychological counselling, mean total episode payment, and regular monitoring of morbidity and mortality. The structure and outcome quality indicator was a combined measure of hospital volume and outcomes^48^. A complete list of unique quality indicators by Donabedian domain is available in *Table S1*. A total of 30 of the 91 (33%) quality indicators identified in this review were endorsed by the NQF, the CMS, or HIS (*Table S1*).

**Table 5:** Characteristics of quality indicators

**Donabedian domain(s)**	
Process	58 (64)
Outcome	26 (29)
Structure	6 (7)
Structure and outcome	1 (1)
Total, *n*	91
**Clinical flow**	
Preoperative	25 (27)
Intraoperative	28 (31)
Postoperative	30 (33)
Preoperative, intraoperative, and postoperative	1 (1)
Preoperative and postoperative	2 (2)
Preoperative and intraoperative	1 (1)
Intraoperative and postoperative	2 (2)
Total, *n*	89*

Values are *n* (%) unless otherwise indicated. *Two innovation indicators not classified.

When grouped by which aspect of the care pathway they evaluated, the quality indicators were nearly evenly distributed among preoperative (25; 27%), intraoperative (28; 31%), and postoperative (30; 33%). Preoperative quality indicators were measures such as completion of preoperative functional assessment, clinical staging, and length of waiting times for surgery. Intraoperative measures included sentinel lymph node biopsy rate, mesorectal excision rate (colon cancer), and risk-adjusted margin positivity rate. Postoperative quality indicators included prolonged length of stay, in-hospital mortality, and readmission rate. A total of six (7%) quality indicators fell into more than one category^28,43,79,80^ (for example patient-reported experience of all care received for a surgical episode) and two (2%) did not fit into any of these groupings as they related to the structure of the healthcare delivery system and/or innovation (for example regular monitoring of morbidity and mortality)^75,80^. A list of quality indicators by clinical flow domain is presented in *Table S2*.

Quality indicators are listed according to QICC domain in *Table S3*, but are not quantified because quality indicators often were not meant to evaluate just one QICC domain and so this grouping was subjective. There were also several quality indicators that spanned more than one QICC domain. These are listed at the bottom of *Table S3*. Quality indicators are also listed by tumour type in *Table S4*.

### Purpose of surgical performance reporting

A total of 43 studies (48%) sought to identify hospital outliers (typically defined as being 2 or 3 standard deviations from the mean), both positive and negative, 29 studies (33%) aimed to provide evidence of variation in practice across hospitals, 14 studies (16%) were interested in which hospitals met a predetermined benchmark for guideline adherence, a certain outcome, or the like, and three studies had other purposes (comparing a single hospital with the national average (1; 1%)^36^, evaluating the effect of adjusting for case-mix variation on hospital ranking (1; 1%)^63^, and proving quality indicator validity by demonstrating the ability to detect variation (1; 1%)^31^) (*Table 6*).

**Table 6:** Study purpose and feedback to studied hospitals/regions

**Purpose of surgical performance reporting**	
Identify outliers	43 (48)
Provide evidence of variation	29 (33)
Compare with benchmark	14 (16)
Other	3 (3)
**Feedback to studied hospitals/regions?**	
Yes	22 (25)
No	67 (75)
**Type of feedback**	
Fed into regional quality improvement	9 (39)
Fed into national quality improvement	6 (27)
Sent to studied hospitals/regions	4 (18)
Sent and fed into quality improvement	3 (13)

Values are *n* (%).

### Risk/case-mix adjustment

Most of the studies (72; 83%) adjusted for case-mix variation in some manner. A few studies (15, 17%) reported not adjusting for patient-level factors^19,20,34–38,43,46,52,53,57,65,73,75,76,81^ and two studies (2%) did not mention whether they made adjustments^71,82^.

While a formal sub-analysis of the variables included in the risk adjustment was not undertaken, most risk-adjusted studies accounted for patient characteristics such as age, sex, race, physical status (for example ASA classification), and co-morbidities (for example Charlson co-morbidity index). Some also adjusted for cancer-related variables such as tumour histological subtype, oncological stage, and neoadjuvant therapy status. A few also included operative factors such as emergent/urgent surgery classification, operating time, and operative approach (typically for outcome measures such as 30-day readmission rate or morbidity rate).

### Intended use and feedback to studied hospitals/regions

Most studies (74; 83%) were intended for academic exploratory use. Aims of exploratory studies included highlighting variation in performance across hospitals on a specific metric, investigating associations between hospital factors and performance on quality indicator(s), and comparing the performance of similar metrics (for example 30- *versus* 90-day mortality rates). The remainder (15; 17%) were descriptive reports of established quality improvement or reporting programmes^24,33,43,44,46,52–54,56,57,60,64–66,71^. These programmes included clinical audits (for example the Dutch Surgical Colorectal Audit) and regional quality improvement programmes (for example the Michigan Surgical Quality Collaborative). See Supplemental Results and Table S5 for further description of data visualization approaches in these studies.

The authors of 22 studies (25%) reported feeding the results back to the groups (that is hospitals or regions) they studied (see *Table S5* for details)^14,21,22,33,37,42–44,46,47,49,50,52,53,57,64,71,73,78,81,83,84^, whereas 67 studies (75%) made no mention of feeding the results back to the hospitals or regions studied. Those authors who did report providing feedback fed the results into quality improvement efforts at the regional (9; 39%) or national (6; 27%) level, sent the results to all studied groups (4; 18%), or both sent the results to all studied groups and fed the results into quality improvement efforts (3; 13%). Notably, only one of the 22 studies advocated for public reporting of their outcomes^39^. The feedback of all other studies was explicitly for the use of healthcare systems.

## Discussion

This analysis sought to describe quality indicators used to compare the quality of surgical oncology care across hospitals or regions. Most quality indicators were process measures and were evenly distributed among preoperative, intraoperative, and postoperative care. The data used to make these comparisons primarily came from national or regional data registries as well as collaborations between specialty societies and not-for-profit organizations. The purpose of evaluating inter-hospital or inter-regional variation fell into one of three main categories: identifying outliers; comparing centres with a predetermined benchmark; or supplying evidence of practice variation. Interestingly, only 22 studies (25%) reported notifying those providing the data of the variation they detected or otherwise using the results to inform quality improvement efforts. Only one study advocated for public reporting of their findings^39^. The majority of these quality indicators were derived from high-income settings.

This review can be utilized to encourage health systems and regions that have already begun employing quality indicators to consider additional previously validated metrics or adapt those that have previously been used. The study team has completed an international Delphi study to establish a minimum set of surgical oncology indicators in all resource settings, including low- and middle-income countries (LMICs)^85^. The present review also provides a framework for healthcare systems interested in starting such initiatives by providing a rigorous overview of the procedures for reporting and feedback, including data sources and governance of reporting programmes. The present review uniquely moves beyond a set of ‘ideal indicators’ that many consensus initiatives have sought to establish to a set of indicators that have been implemented or tested for validity and fairness. The review also exposes the gaps in current metrics, with a paucity of outcome measures to appraise surgical oncology care used, the need for integrated data architectures, and the routine collection of patient-reported outcome measures (PROMs) all providing opportunities for improving care.

Only 29% of quality indicators in the surgical oncology literature were outcome indicators. This is likely because processes are proxies that are easier to measure than outcomes, which are subject to issues such as loss to follow-up and case-mix variation. Many process indicators were highly specific to tumour-type specialty guidelines. However, some indicators were tumour agnostic (for example whether an adequate lymphadenectomy had been performed). Likewise, achievement of negative margins was measured in several ways, including risk-adjusted margin positivity (RAMP) rate, rate of resections with a negative margin (that is R0 resections), and circumferential margin positivity rate. Another theme that emerged was proxy measurement of overtreatment. Two examples of these measures were the rate of radical mastectomy for pre-invasive breast cancer and the proportion of prostate biopsies in men with limited life expectancy. Several studies compared 30- and 90-day mortality/readmission/reoperation rates, suggesting ongoing disagreements regarding the validity of some outcome measures.

Composite measures identified in this review included ‘textbook outcome’ (a binary indicator achieved when a patient had the ideal outcome as defined by a group of experts), MTL22 (‘mortality, transfer, or prolonged length of stay in first 22 postoperative days’), and a combined measure of volume and outcome^33,36,48,77^. The purported benefit of composite measures is their superior sensitivity as they capture related outcomes.

It is noteworthy that no studies compared PROMs. This might be a factor of limited collection of PROMs, difficulty linking this information to patient records, and/or belief that these measures are less reliable or valid despite evidence demonstrating concordance between PROMs and clinician-reported outcome measures^86^. Additionally, measures of access and innovation/improvement were absent. However, collection of these quality indicators is feasible as demonstrated by national audits such as the National Prostate Cancer Audit in the UK^87^.

Clinically meaningful quality indicators need to be valid, feasible to collect, have clinician ‘buy-in’, and show variation over space and time^10^. Each of these components holds challenges. Validating a quality indicator includes ensuring differences in an indicator are reflective of true differences in quality^88^. Validity was typically demonstrated a priori for these quality indicators by an academic study and/or specialty society consensus. Even still, reporting comparative quality indicator performance in a thoughtful manner that is fair (that is adjusted for case-mix variation and volume) and understandable by the public is challenging^89^.

Feasibility of data collection can also pose a challenge. This is attributable in part to barriers to routine performance measurement such as lack of a shared data structure, consistent means of patient identification, and assurance of data completeness, particularly in decentralized healthcare systems such as in the USA^90^. Existing administrative data sets, while less burdensome, may be missing clinically important information such as co-morbidities that are necessary for case-mix adjustment. Purpose-made data sets for quality indicator comparison may be more complete, but with the trade-off of additional burden on clinicians or staff members for data collection and entry, which can impact on completeness.

National clinical data registries were by far the most common source of data (64; 72%), while collaborations between specialty societies and not-for-profit organizations (for example the American Cancer Society) (24; 27%) and specialty societies alone (15; 17%) were the most common entities that produced/maintained databases.

Acknowledging the expectation that many studies would be exploratory, perhaps the most notable result of this analysis is that only 22 (25%) of the included studies reported feeding the results back to those who provided the data or using them to inform quality improvement efforts. Of these, only one advocated for public reporting of their results^39^. The other 67 studies were performed purely for academic purposes.

If the ultimate aim of measuring and comparing quality is to improve quality, it is worth considering how results of these analyses could be used to that end. Berwick *et al.*^91^ proposed two pathways for quality improvement based on the intrinsic motivation of providers (change pathway) or through market competition whereby patients select a provider of their choice. Feeding the results back to those who provided the data for these studies and using the results to inform quality improvement efforts both rely on hospitals’ intrinsic motivation to improve their care delivery^91–93^. While some healthcare organizations may be responsive to private communication of comparative results, evidence shows that public reporting of hospital performance spurs significantly more quality improvement activities^94,95^. Several systematic reviews with meta-analyses have been published that show the opposite effect^95–97^. Even at an individual clinician level, a 2018 study by Vallance *et al*.^98^ found that surgeon-specific outcome reporting in colorectal cancer surgery did not lead to gaming or adverse clinical practices, but did lead to a significant reduction in 90-day mortality.

Additionally, there are now multiple examples of successful public reporting initiatives^87^. The UK Bowel Cancer Audit, for instance, has shown a reduction in 90-day and 2-year mortality, shrunk the variation between providers in use of adjuvant chemotherapy and 30-day readmissions, and increased the proportion using laparoscopic surgery between 2015 and 2019^99^. The evidence demonstrates that public reporting is feasible and has the potential to improve the quality of patient care. However, this requires buy-in from stakeholders, including clinicians and health system administrators, and investment in integrated data systems.

If comparative quality measures are available to those who choose where care is received (that is public reporting), market competition may take its course. Because lower performing hospitals stand to lose revenue, they will be incentivized to improve their quality to attract more patients. New York state, for example, has been publicly reporting short-term cardiac surgery outcomes for over 20 years and many hospitals, concerned about losing market share, have undertaken quality improvement initiatives as a result^95^.

Evidence suggests patients are prepared to travel to alternative, more distant elective secondary care services, but tend to be more sensitive to hospital-level characteristics (for example availability of robotic surgery and overall hospital quality) than to cancer-specific outcome measures^100,101^. This is likely driven in part by the strength of informal knowledge networks, which tend to focus on the brand and reputation of providers, as well as the relevance of current cancer-specific outcomes when patients consider their own care choices. Further patient engagement will be critical to support rational use of metrics^102^.

All studies included in this article were conducted in high-income countries (HICs). This is notable because the context within which a health system operates effects not only how and what care is provided but also dictates what quality indicators are (or can be) collected. For example, though each HIC has a unique health system structure, the infrastructure available to support data collection and analysis is more uniformly robust than that in their LMIC counterparts.

The largest burden of cancer in the next few years will be in emerging economies and LMICs^2^. This review shows a paucity of published measures in this setting. Anecdotally, there is heterogeneity in quality indicators collected in these settings, underscoring a need to develop basic matrices that can be used in diverse settings to ensure the quality of surgical oncology services. This study can be used to support LMICs in selecting quality measures to deploy in their own healthcare systems by providing a menu of validated surgical oncology indicators.

It is notable that 15 studies did not adjust for case-mix variation in their analyses (and 2 studies did not mention whether they did). Risk adjustment is important to ensure the validity of results and to prevent cream skimming/gaming. Without risk adjustment, a hospital that cares for sicker patients (that is more co-morbidities and higher stage at diagnosis) may appear to have a poorer performance than one that treats fewer complex patients, even if they have the same true level of quality (that is given the same patients they would perform the same). This can frustrate the hospitals/regions being compared and cause them to distrust the results and lose confidence in the process. Alternatively, the hospitals/regions might engage in cream skimming or gaming whereby they selectively provide care to fewer complex patients to inflate their performance^98,103^.

There are limitations to the analyses presented in this review that should be acknowledged. First, only published inter-hospital and inter-regional quality comparisons could be reviewed. There are likely quality comparisons undertaken for internal audits or other purposes that are not published and so were not included in this review. Embase and MEDLINE were used to ensure that a wide range of published papers was included, but it is acknowledged that some regional country-specific initiatives may not have been included if they were not published or translated into English. Additionally, the categorization of the indicators into QICC domains was not always simple. While uncertainties were resolved via discussion with two subject matter experts, there was an unavoidable element of judgement required in these decisions. For this reason, two outcome-related QICC categories were combined and this review did not quantify the number of indicators in each QICC category, but rather listed the indicators in the category that were judged most appropriate. Lastly, only studies that had full-text availability in English were included.

There is a modest corpus of comparisons of quality indicators in surgical oncology almost entirely drawn from HICs such as the USA, the Netherlands, and the UK. These comparisons can be used to improve quality in the healthcare system by providing feedback to those providing the data via sharing the results or undertaking evidence-informed quality improvement efforts and/or by providing useable information about detected differences in quality to aid decisions about where to receive care and to inspire public trust. However, substantial gaps exist, especially in developing quality indicators for surgical oncology that are relevant across different health systems.

Once this core set of quality indicators is identified, organizations that collect quality-related data might consider generating performance reports to be shared with leaders of hospitals and/or regions whose data they collect. These same organizations could also facilitate knowledge sharing among participating hospitals/regions to improve quality across the board. Defining a core indicator set may also open the door to broader comparisons across data sets and health systems. Tiering the indicator set based upon the robustness of the data and health system infrastructure could aid prioritization of quality improvement efforts in developing health systems.

## Supplementary Material

zrae009_Supplementary_Data

## Data Availability

Data available upon request.
