# Supplementary material for: Quality indicators in surgical oncology: systematic review of measures used to compare quality across hospitals
Source: BJS Open. 2024 Mar 21;8(2):zrae009. doi: 10.1093/bjsopen/zrae009 (PMC10957165; doi:10.1093/bjsopen/zrae009)
Supplement: zrae009_Supplementary_Data [file zrae009_supplementary_data.docx]

**Quality Indicators in Surgical Oncology: systematic review of measures used to compare quality across hospitals**

Megan McLeod, MD, MSc^1,2^, Kari Leung, MPharm, MSc^3^, CS Pramesh, MS, FRCS^4^, Peter Kingham, MD, FACS^5^, Miriam Mutebi, MD^6^, Julie Torode, PhD^7^, Andre Ilbawi, MD^8^, Jade Chakowa^9^, Richard Sullivan, FRCS, PhD^10^, Ajay Aggarwal, MD, PhD^11^

1. London School of Economics and Political Science, London, UK
2. Department of Otolaryngology – Head & Neck Surgery, Vanderbilt University Medical Center, Nashville, TN, USA
3. Guy’s & St Thomas’ NHS Trust, London, UK
4. Tata Memorial Centre, Homi Bhabha National Institute, Mumbai, India
5. Department of Surgery, Memorial Sloan Kettering Cancer Center, New York, New York, USA
6. Department of Surgery, Aga Khan University, Nairobi, Kenya
7. Institute of Cancer Policy, Centre for Cancer, Society & Public Health, King’s College London, UK
8. World Health Organisation, Geneva, Switzerland
9. City Cancer Challenge, Geneva, Switzerland
10. Institute of Cancer Policy, Global Oncology Group, Centre for Cancer, Society & Public Health, King’s College London, UK
11. London School of Hygiene and Tropical Medicine, London, UK

Corresponding author:

Megan McLeod

1211 Medical Center Drive

Nashville, TN 37232

[Megan.mcleod@vumc.org](mailto:Megan.mcleod@vumc.org)

+1 303-993-9944

ORCID ID – 0000000262905310

Twitter: @Megan_McLeod_

**Supplementary Materials - Index**

| **Supplementary Results** | |  | |
| --- | --- | --- | --- |
| *Presentation/visualization of data* | | *page 3* | |
| **Supplementary Appendixes** | |  | |
| *Search Terms* | | *page 4* | |
| **Supplementary Tables and Figures** | |  | |
| *Supplemental Figure 1* | | *page 5* | |
| *Supplemental Table 1* | | *page 6* | |
| *Supplemental Table 2* | | *page 11* | |
| *Supplemental Table 3* | | *page 16* | |
| *Supplemental Table 4* | | *page 21* | |
| *Supplemental Table 5* | | *page 28* | |
| *Supplemental Table 6* | | *page 75* | |
|  | |  | |
|  | |  | |
|  | |  | |
|  | |  | |
|  | |  | |
|  | |  | |

**Supplementary Results**

## *Presentation/visualization of data*

Data was visualized in a variety of ways (Supplemental Table 6). The most common (n = 28, 31%) means of graphically identifying outliers or comparing centers to a pre-determined benchmark was via a scatter chart with a superimposed benchmark. For outlier studies, positive outliers could be seen above the top “guard rail” representing a certain number of standard deviations from the mean (typically two or three) while negative outliers fell below the lower “guard rail”. For benchmark studies, hospitals or regions exceeding the benchmark could be seen above the benchmark cutoff (typically represented by a dashed horizontal line) and those falling short were plotted below the benchmark cutoff. When the benchmark was not pre-determined, authors used the mean to represent the “typical” value for a given QI. Twenty-one (24%) studies made similar graphic comparisons using a bar chart with a benchmark (pre-determined or using the mean). Eighteen (20%) studies made use of a funnel plot which superimposed “guard rails” that were wider for centers with smaller volumes and narrowed as the volumes increased. This allowed identification of outliers while accounting for the fact that smaller sample size is subject to greater variation. Other methods of visualizing data included bar charts without benchmarks (n = 5, 6%), Kaplan-Meier curves showing performance of positive and negative outliers versus the average (n = 5, 6%), trendlines of percentage meeting a QI (n = 4, 4%), and heatmaps (n = 1, 1%). Ten studies (11%) did not present results graphically, favoring the sole use of tables.

**Supplementary Appendixes**

## ***Search Terms***

Embase final search terms:

(quality of healthcare.ti. or quality of healthcare.ab. or quality improvement.ab. or quality improvement.ti. or quality indicator.ab. or quality indicator.ti. or quality measure.ab. or quality measure.ti. or performance indicator.ti. or performance indicator.ab. or performance measure.ab. or performance measure.ti. or outcome indicator.ab. or outcome indicator.ti. or outcome measure.ab. or outcome measure.ti. or outcome metric.ti. or outcome metric.ab. or quality metric.ti. or quality metric.ab. or performance metric.ab. or performance metric.ti.) AND (surgery.ti. or surgery.ab. or surgical.ab. or surgical.ti.) AND (cancer.ti. or cancer.ab. or neoplasm.ab. or neoplasm.ti. or tumor.ab. or tumor.ti. or tumour.ab. or tumour.ti. or oncology.ti. or oncology.ab.) AND (limit to (human and English language and yr="2000 - 2023") and (article or article in press or books or chapter) and (adult <18 to 64 years> or aged <65+ years>))

MEDLINE final search terms:

((quality of healthcare[Title/Abstract]) OR (quality improvement[Title/Abstract]) OR (quality measure[Title/Abstract]) OR (quality metric[Title/Abstract]) OR (performance measure[Title/Abstract]) OR (performance metric[Title/Abstract]) OR (outcome measure[Title/Abstract]) OR (outcome metric[Title/Abstract]) OR (quality indicator[Title/Abstract]) OR (performance indicator[Title/Abstract]) OR (outcome indicator[Title/Abstract])) AND ((surgery[Title/Abstract]) OR (surgical[Title/Abstract])) AND ((cancer[Title/Abstract]) OR (neoplasm[Title/Abstract]) OR (tumor[Title/Abstract]) OR (tumour[Title/Abstract]) OR (oncology[Title/Abstract]))

**Supplementary Figures and Tables**

1. b.

c. d.

# **Supplemental Figure 1. Histograms of number of hospitals and patients evaluated**

This figure demonstrates the density of studies by the number of hospitals they compared and the number of patients they included. Panels a. and c. show the total number of hospitals evaluated. Panel c. shows the distribution of the density of studies in the densest part of Panel a. (0-100 hospitals). Panels b. and d. show the distribution of the total number of patients studied. Panel d. shows the distribution of the density of studies in the densest part of Panel b. (0-10,000 patients).

|  | **Specific Indicators** | **No.** | **(%)** |  |
| --- | --- | --- | --- | --- |
| **Donabedian Domain(s)** |  |  |  |  |
| Process |  | 58 | (64) |  |
|  | Adequate follow-up (2+ follow-up office visits within 1 year of treatment completion) |  |  |  |
|  | Average number of follow-up visits per patient |  |  |  |
|  | Average time from initial excision to definitive surgery |  |  |  |
|  | Breast conserving surgery (BCS) rate |  |  |  |
|  | Carcinoembryonic antigen (CEA) obtained |  |  |  |
|  | Clinical staging performed |  |  | *NQF |
|  | Core needle biopsy rate in breast cancer |  |  | *NQF |
|  | Incidence of repeat biopsy |  |  |  |
|  | Insufficient surgical margins |  |  | *HIS |
|  | Median number of days between first colonoscopy and first resection |  |  |  |
|  | Mesorectal excision rate |  |  |  |
|  | Circumferential resection margin positivity |  |  | *HIS |
|  | Odds of surgical resection of early small cell lung cancer |  |  | *HIS |
|  | Patient reported experience |  |  |  |
|  | Percentage of node negative patients who underwent axillary dissection |  |  | *NQF |
|  | Percentage of patients achieving negative margins |  |  | *HIS |
|  | Percentage of women with 'unknown' margin status |  |  |  |
|  | Pretherapy functional assessment completed |  |  | *NQF |
|  | Proportion of adequate lymphadenectomies |  |  | *NQF |
|  | Proportion of prostate biopsies performed for men with limited life expectancy |  |  |  |
|  | Proportion of axillary staging by sentinel lymph node biopsy (SLNB) only in pN0 |  |  |  |
|  | Proportion of cases for which 1) T1a tumors underwent partial nephrectomy and 2) T1-T2 tumors underwent minimally invasive radical nephrectomy |  |  | *HIS |
|  | Proportion of colorectal patients who have pre-operative chest X-ray and abdominal ultrasound, CT scan or MRI |  |  |  |
|  | Proportion of colorectal patients who have undergone surgical resection for colon or rectal cancer have documentation that colonoscopy or barium enema with sigmoidoscopy was offered within 6 months before or after surgery |  |  | *NQF |
|  | Proportion of ductal carcinomina in-situ (DCIS) patients receiving no axillary dissection |  |  |  |
|  | Proportion of Non-small cell lung cancer (NSCLC) patients who had FEV1 (forced expiratory volume) and DLCO (diffusing capacity of lungs with carbon monoxide) performed before surgery |  |  | *NQF |
|  | Proportion of patients discussed in multi-disciplinary team (MDT) meeting within 6 weeks after incidence date |  |  | *HIS |
|  | Proportion of patients provided uniformly guideline-concordant care |  |  |  |
|  | Proportion of patients receiving total thyroidectomy for papillary cancer>1.5 cm or node positive |  |  |  |
|  | Proportion of patients treated via conventional excision versus Mohs micrographic surgery (MMS) |  |  |  |
|  | Proportion of patients undergoing central neck dissection for node-positive papillary thyroid cancer |  |  |  |
|  | Proportion of patients undergoing mammogram within 14 months of definitive surgery for breast conservation or unilateral mastectomy |  |  |  |
|  | Proportion of patients who are diagnosed with colon cancer and do not have metastatic disease were offered a curative resection within 6 weeks of diagnosis. (Separating patients with diagnosis and treatment of care in the same hospital vs multiple sites) |  |  |  |
|  | Proportion of patients who are diagnosed with rectal cancer that appears clinically to be Stage II or III, and was offered surgical resections within 16 weeks after beginning of concurrent chemoradiotherapy (CCRT) |  |  |  |
|  | Proportion of papillary thyroid carcinoma tumors <4cm resected via total thyroidectomy |  |  |  |
|  | Proportion of resections completed via laparoscopy/minimally invasive approach |  |  |  |
|  | Proportion of surgeries for cancers <10mm where frozen section is NOT performed |  |  |  |
|  | Proportion of T1a tumors undergoing partial nephrectomy. |  |  | *HIS |
|  | Proportion of waiting times for surgery from first diagnostic test <42 days |  |  |  |
|  | Proportion of waiting times for surgery from referral ≤30 days |  |  |  |
|  | Proportion of waiting times for surgery from screening test <60 days and <90 days |  |  |  |
|  | Rate of accuracy between cN and pN status in lung cancer |  |  |  |
|  | Rate of anatomic resection for T1b or greater |  |  |  |
|  | Rate of complete axillary lymph node dissection (CALND) for node positive SLNB |  |  |  |
|  | Rate of circumferential resection margin (CRM) ‘missingness’ |  |  | *NQF |
|  | Rate of fine needle aspiration in thyroid cancer |  |  |  |
|  | Rate of invasive mediastinal staging (IMS) in lung cancer |  |  |  |
|  | Rate of lymph node sampling at the time of resection |  |  |  |
|  | Rate of preoperative ostomy site marking by ostomy nurse |  |  |  |
|  | Rate of receipt of curative-intent surgery |  |  | *HIS |
|  | Rate of sentinel lymph node biopsy |  |  | *NQF |
|  | Rate of use of abdominoperineal excision (APE) |  |  |  |
|  | Risk-adjusted margin positivity rate (RAMP) |  |  |  |
|  | Rate of SLNB in cN0 patients |  |  |  |
|  | Sphincter-preserving surgery rate |  |  |  |
|  | Surgery within 5 weeks of diagnosis |  |  |  |
|  | Treatment options and risks discussed |  |  |  |
|  | Treatment within 60 days of diagnosis |  |  |  |
| Outcome |  | 26 | (29) |  |
|  | 30-day complication rate |  |  | *NQF |
|  | 30-day morbidity rate |  |  |  |
|  | 30-day mortality |  |  | *NQF |
|  | 30-day or in hospital mortality rate |  |  | *NQF |
|  | 30-day readmission rate |  |  | *NQF |
|  | 5-year overall survival |  |  |  |
|  | 90-day mortality |  |  | *HIS |
|  | 90-day readmission rate |  |  |  |
|  | Composite of death or at least one of the following serious morbidities (DSM) occurring within 30 days |  |  |  |
|  | Complicated course rate (any complication combined with a prolonged length  of hospital stay (>14 days), and/or complication requiring surgical, endoscopic, or radiological intervention, and/or any complication combined with prolonged intensive care unit stay (>1 day), and/or death within 30 days after the procedure, and/or death during hospital admission following surgery.) |  |  |  |
|  | Failure to cure |  |  |  |
|  | Failure-to-rescue rate |  |  | *NQF |
|  | In-hospital mortality |  |  | *NQF |
|  | Inability to receive planned chemotherapy |  |  |  |
|  | Length of stay |  |  | *NQF |
|  | MTL22 - (a) mortality and/or (b) transfer to another hospital (other than rehabilitation clinic) within 22 days after the index operation, and/or (c) postoperative length of hospital stay≥22 days. |  |  |  |
|  | MTL30 - (a) mortality and/or (b) transfer to another hospital (other than rehabilitation clinic) within 30 days after the index operation, and/or (c) postoperative length of hospital stay≥30 days. |  |  |  |
|  | Occurrence of Agency for Healthcare Research and Quality (AHRQ) patient safety indicators (PSIs) -- secondary infections, postoperative DVT/PE (deep vein thrombosis/pulmonary embolism), wound dehiscence |  |  | *AHRQ |
|  | Percentage of re-resections following breast conserving surgery (BCS) |  |  | *HIS |
|  | Proportion of NSCLC patients who died within 60 days after primary surgery |  |  |  |
|  | Rate of anastomotic leakage (AL) |  |  | *HIS |
|  | Rate of ipsilateral breast tumor recurrence (IBTR) |  |  |  |
|  | Reexcision rates for initial negative margins |  |  |  |
|  | Reoperation rate |  |  | *NQF |
|  | Textbook outcome rate (Ovarian) |  |  |  |
|  | Textbook outcome rate (Pancreatic) |  |  |  |
| Structure |  | 6 | (7) |  |
|  | Average total episode payment (for 3 procedures) |  |  |  |
|  | Center employs at least one board-certified urologist |  |  |  |
|  | Involvement in research |  |  |  |
|  | Maintenance of a local thoracic surgery database or participate in a national or international database |  |  | *NQF |
|  | Regular monitoring of morbidity and mortality |  |  |  |
|  | Psychological counseling available |  |  |  |
| Structure; Outcome |  | 1 | (1) |  |
|  | CM-V&O (combined measure of volume & outcome) |  |  |  |

**Supplemental Table 1. Quality Indicators by Donabedian Domain**

Abbreviations: cN = clinical nodal stage, pN = pathologic nodal stage

|  | | | |
| --- | --- | --- | --- |
|  | **Quality Indicator** | **No.** | **(%)** |
| **Clinical Flow Domain** |  |  |  |
| Preoperative |  | 25 | (27) |
|  | Carcinoembryonic antigen (CEA) obtained |  |  |
|  | Treatment within 60 days of diagnosis |  |  |
|  | Proportion of patients who are diagnosed with colon cancer and do not have metastatic disease were offered a curative resection within 6 weeks of diagnosis. (Separating patients with diagnosis and treatment of care in the same hospital vs multiple sites) |  |  |
|  | Proportion of patients who are diagnosed with rectal cancer that appears clinically to be Stage II or III, and was offered surgical resections within 16 weeks after beginning of CCRT |  |  |
|  | Average time from initial excision to definitive surgery |  |  |
|  | Proportion of patients receiving Total thyroidectomy for papillary cancer>1.5 cm or node positive |  |  |
|  | Rate of preoperative ostomy site marking |  |  |
|  | Proportion of waiting times for surgery from first diagnostic test <42 days |  |  |
|  | Proportion of waiting times for surgery from screening test <60 days and <90 days |  |  |
|  | Median number of days between first colonoscopy and first resection |  |  |
|  | Pretherapy functional assessment completed |  |  |
|  | Treatment options and risks discussed |  |  |
|  | Surgery within 5 weeks of diagnosis. |  |  |
|  | Proportion of NSCLC patients who had FEV1 and DLCO performed before surgery |  |  |
|  | Proportion of all biopsies performed for men with limited LE |  |  |
|  | Clinical staging performed |  |  |
|  | Core needle biopsy rate |  |  |
|  | Incidence of repeat biopsy |  |  |
|  | Proportion of colorectal patients who have pre-operative chest X-ray and abdominal ultrasound, CT scan or MRI |  |  |
|  | Proportion of waiting times for surgery from referral ≤30 days |  |  |
|  | At least one board-certified urologist |  |  |
|  | Rate of receipt of curative-intent surgery |  |  |
|  | Rate of fine needle aspiration |  |  |
|  | Proportion of patients discussed in MDT within 6 weeks after incidence date |  |  |
|  | Odds of surgical resection (O:E) |  |  |
| Intraoperative |  | 28 | (31) |
|  | Sphincter-preserving surgery (SPS) rate |  |  |
|  | Proportion of patients with 1) T1a tumors undergoing partial nephrectomy and 2) T1-T2 tumors undergoing minimally invasive radical nephrectomy. |  |  |
|  | Percentage of women with 'unknown' margin status |  |  |
|  | Breast-conserving surgery (BCS) rate |  |  |
|  | Insufficient surgical margins |  |  |
|  | Seninel lymph node biopsy (SLNB) in cN0 patients |  |  |
|  | Proportion of patients provided uniformly guideline-concordant care |  |  |
|  | Proportion of patients undergoing central neck dissection for node-positive papillary thyroid cancer |  |  |
|  | Mesorectal excision rate |  |  |
|  | Rate of lymph node sampling at the time of resection |  |  |
|  | Rate of anatomic resection for T1b or greater |  |  |
|  | Rate of SLNB |  |  |
|  | Rate of complete axillary lymph node dissection (CALND) for node positive SLNB |  |  |
|  | Rate of CRM ‘missingness’ |  |  |
|  | Proportion of T1a tumors undergoing partial nephrectomy. |  |  |
|  | Risk-adjusted margin positivity rate (RAMP) |  |  |
|  | Proportion of adequate lymphadenectomies |  |  |
|  | Rate of use of abdominoperineal excision (APE) |  |  |
|  | Observed:expected rate of circumferential resection margin positivity |  |  |
|  | Proportion of surgeries for cancers <10mm where frozen section is NOT performed |  |  |
|  | Proportion of axillary staging by SLNB only in pN0 |  |  |
|  | Proportion of ductal carcinoma in situ (DCIS) patients receiving no axillary dissection |  |  |
|  | Percentage of node negative patients who underwent axillary dissection |  |  |
|  | Proportion of resections completed via laparoscopy/minimally invasive approach |  |  |
|  | Percentage of patients achieving negative margins |  |  |
|  | Rate of invasive mediastinal staging (IMS) |  |  |
|  | Proportion of patients treated via conventional excision versus Mohs micrographic surgery (MMS) |  |  |
|  | Proportion of PTC tumors <4cm resected via total thyroidectomy |  |  |
| Postoperative |  | 30 | (33) |
|  | 30-day morbidity rate |  |  |
|  | Adjusted 30-day mortality |  |  |
|  | Adjusted 90-day mortality |  |  |
|  | Complicated course rate |  |  |
|  | Inability to receive planned chemotherapy |  |  |
|  | Reoperation rate |  |  |
|  | Length of stay |  |  |
|  | Failure-to-rescue rate |  |  |
|  | Occurrence of Agency for Healthcare Research and Quality (AHRQ) patient safety indicators (PSIs) -- secondary infections, postoperative DVT/PE (deep veing thrombosis/pulmonary embolism), wound dehiscence |  |  |
|  | 90-day readmission rate |  |  |
|  | MTL22 - (a) mortality and/or (b) transfer to another hospital (other than rehabilitation clinic) within 22 days after the index operation, and/or (c) postoperative length of hospital stay≥22 days. |  |  |
|  | MTL30 - (a) mortality and/or (b) transfer to another hospital (other than rehabilitation clinic) within 30 days after the index operation, and/or (c) postoperative length of hospital stay≥30 days. |  |  |
|  | In-hospital mortality |  |  |
|  | Monitor morbidity and mortality regularly |  |  |
|  | Proportion of patients undergoing mammogram within 14 months of definitive surgery for breast conservation or unilateral mastectomy |  |  |
|  | 30-day complication rate |  |  |
|  | Composite of death or at least one of the following serious morbidities (DSM) occurring within 30 days |  |  |
|  | 30-day adjusted readmission rate |  |  |
|  | 5-year overall survival |  |  |
|  | Reexcision rates for initial negative margins |  |  |
|  | Rate of ipsilateral breast tumor recurrence (IBTR) |  |  |
|  | Rate of anastomotic leakage (AL) |  |  |
|  | Adequate follow-up (2+ follow-up office visits within 1 year of treatment completion) |  |  |
|  | Percentage of re-resections following breast conserving surgery (BCS) |  |  |
|  | 30-day or in hospital mortality rate after parenchymal lung resection |  |  |
|  | Average number of follow-up visits per patient |  |  |
|  | Textbook outcome rate (Ovarian) |  |  |
|  | Textbook outcome rate (Pancreatic) |  |  |
|  | Failure to cure |  |  |
|  | Proportion of non-small cell lung cancer (NSCLC) patients who died within 60 days after primary surgery |  |  |
| Preoperative; Intraoperative; Postoperative |  | 1 | (1) |
|  | Patient reported experience |  |  |
| Preoperative; Postoperative |  | 2 | (2) |
|  | Proportion of colorectal patients who have undergone surgical resection for colon or rectal cancer have documentation that colonoscopy or barium enema with sigmoidoscopy was offered within 6 months before or after surgery |  |  |
|  | Psychological counseling available |  |  |
| Preoperative: Intraoperative |  | 1 | (1) |
|  | Rate of accuracy between cN and pN status |  |  |
| Intraoperative; Postoperative |  | 2 | (2) |
|  | Average total episode payment (for 3 procedures) |  |  |
|  | CM-V&O (combined measure of volume & outcome) |  |  |
| Total |  | 89 |  |

*2 innovation indicators not classified

**Supplemental Table 2. Quality Indicators by Clinical Flow Domain**

Abbreviations: cN = clinical nodal stage, pN = pathologic nodal stage

|  | |
| --- | --- |
|  | **Specific Indicators** |
| **Quality of Cancer Care Domain** |  |
| Healthcare Delivery System |  |
|  | Center employs at least one board-certified urologist |
| Access |  |
|  | Average total episode payment (for 3 procedures) |
|  | Psychological counseling available |
| Timeliness |  |
|  | Treatment within 60 days of diagnosis |
|  | Average time from initial excision to definitive surgery |
|  | Proportion of waiting times for surgery from referral ≤30 days |
|  | Proportion of waiting times for surgery from first diagnostic test <42 days |
|  | Proportion of waiting times for surgery from screening test <60 days and <90 days |
|  | Median number of days between first colonoscopy and first resection |
|  | Surgery within 5 weeks of diagnosis. |
| Appropriateness of care/clinical practice guideline adherence | |
|  | Proportion of all biopsies performed for men with limited life expectancy |
|  | Clinical staging performed |
|  | Carcinoembryonic antigen (CEA) obtained |
|  | Core needle biopsy rate of breast cancer patients (versus excisional biopsy) |
|  | CRM ‘missingness’ rate |
|  | Incidence of repeat biopsy |
|  | Monitor morbidity and mortality regularly |
|  | Proportion of colorectal patients who have pre-operative chest X-ray and abdominal ultrasound, CT scan or MRI |
|  | Proportion of colorectal patients who have undergone surgical resection for colon or rectal cancer have documentation that colonoscopy or barium enema with sigmoidoscopy was offered within 6 months before or after surgery |
|  | Breast Conserving Surgery (BCS) rate |
|  | Sentinel Lymph Node biopsy in cN0 patients |
|  | Proportion of patients provided uniformly guideline-concordant care |
|  | Proportion of patients receiving total thyroidectomy for papillary cancer>1.5 cm or node positive |
|  | Proportion of patients undergoing central neck dissection for node-positive papillary thyroid cancer |
|  | Rate of lymph node sampling at the time of resection |
|  | Rate of anatomic resection for T1b or greater |
|  | Rate of sentinel lymph node biopsy (SLNB) |
|  | Rate of complete axillary lymph node dissection (CALND) for node positive SLNB |
|  | Proportion of patients undergoing mammogram within 14 months of definitive surgery for breast conservation or unilateral mastectomy |
|  | Proportion of T1a renal tumors undergoing partial nephrectomy. |
|  | Proportion of adequate lymphadenectomies |
|  | Proportion of surgeries for cancers <10mm where frozen section is NOT performed |
|  | Proportion of breast cancer patients with ductal carcinoma in situ (DCIS) receiving no axillary dissection |
|  | Percentage of node negative patients who underwent axillary dissection |
|  | Adequate follow-up (2+ follow-up office visits within 1 year of treatment completion) |
|  | Rate of receipt of curative-intent surgery |
|  | Rate of invasive mediastinal staging (IMS) for lung cancer patients |
|  | Rate of fine needle aspiration of thyroid cancers |
|  | Average number of follow-up visits per patient |
|  | Proportion of non-small cell lung cancer (NSCLC) patients who had FEV1 and DLCO performed before surgery |
|  | Odds of surgical resection of early small cell lung cancer |
|  | Proportion of papillary thyroid carcinoma tumors <4cm resected via total thyroidectomy |
|  | Pretherapy functional assessment completed |
| Technical Aspects |  |
|  | Sphincter-preserving surgery rate |
|  | Percentage of women with 'unknown' margin status |
|  | Insufficient surgical margins |
|  | Risk-adjusted margin positivity rate (RAMP) |
|  | Circumferential resection margin positivity |
|  | Proportion of axillary staging by SLNB only in pN0 |
|  | Proportion of resections completed via minimally invasive surgery/laparoscopy |
|  | Percentage of patients achieving negative margins |
|  | Rate of concordance between cN and pN status |
|  | Percentage of re-resections following BCS |
|  | Percentage requiring re-resection following initial negative margins |
|  | Proportion of patients treated via conventional excision versus Mohs micrographic surgery (MMS) |
| Multidisciplinary/coordinated care | |
|  | Rate of preoperative ostomy site marking by ostomy nurse |
|  | Proportion of patients discussed in Multidisciplinary Tumor Board within 6 weeks after incidence date |
| Patient experience/satisfaction | |
|  | Patient reported experience |
|  | Treatment options and risks discussed |
| Disease-specific outcomes\| Safety errors and adverse events | |
|  | 30-day morbidity rate |
|  | Adjusted 30-day mortality |
|  | Adjusted 90-day mortality |
|  | 5-year overall survival |
|  | Complicated course rate |
|  | Rate of ipsilateral breast tumor recurrence (IBTR) |
|  | 30-day or in hospital mortality rate after parenchymal lung resection |
|  | Proportion of NSCLC patients who died within 60 days after primary surgery |
|  | Reoperation rate |
|  | Failure-to-rescue rate |
|  | Occurrence of Agency for Healthcare Research and Quality (AHRQ) patient safety indicators (PSIs) -- secondary infections, postoperative DVT/PE, wound dehiscence |
|  | 90-day readmission rate |
|  | 30-day complication rate |
|  | 30-day readmission rate |
|  | Rate of anastomotic leakage (AL) |
|  | Inability to receive planned chemotherapy |
|  | MTL22 - (a) mortality and/or (b) transfer to another hospital (other than rehabilitation clinic) within 22 days after the index operation, and/or (c) postoperative length of hospital stay≥22 days. |
|  | MTL30 - (a) mortality and/or (b) transfer to another hospital (other than rehabilitation clinic) within 30 days after the index operation, and/or (c) postoperative length of hospital stay≥30 days. |
|  | In-hospital mortality |
|  | Composite of death or at least one of the following serious morbidities (DSM) occurring within 30 days |
|  | Textbook outcome rate (Ovarian) |
|  | Textbook outcome rate (Pancreatic) |
|  | Failure to cure |
| Patient-reported outcomes |  |
|  | None |
| Innovation and improvement in care | |
|  | None |
| (Indicators spanning more than 1 QICC domain) | |
| Appropriateness&Technical |  |
|  | Mesorectal excision rate |
|  | Rate of use of abdominoperineal excision (APE) |
| Appropriateness&Access |  |
|  | Proportion of cases for which 1) T1a tumors underwent partial nephrectomy and 2) T1-T2 tumors underwent minimally invasive radical nephrectomy |
| Appropriateness&Timeliness |  |
|  | Proportion of patients who are diagnosed with colon cancer and do not have metastatic disease were offered a curative resection within 6 weeks of diagnosis. (Separating patients with diagnosis and treatment of care in the same hospital vs multiple sites) |
|  | Proportion of patients who are diagnosed with rectal cancer that appears clinically to be Stage II or III, and were offered surgical resections within 16 weeks after beginning of concurrent chemoradiotherapy (CCRT) |
| Healthcare delivery system&Disease-specific outcomes | |
|  | CM-V&O (combined measure of volume & outcome) |
|  | Average length of stay/prolonged length of stay |
| Healthcare delivery system&Innovation | |
|  | Involvement in research |
|  | Maintenance of a local thoracic surgery database or participate in a national or international database |

**Supplemental Table 3. Quality Indicators by Quality of Cancer Care Domain**

Abbreviations: cN = clinical nodal stage, pN = pathologic nodal stage, DVT = deep vein thrombosis, PE = pulmonary embolism, FEV1 = forced expiratory volume in 1 second, DLCO = diffusing capacity of lungs for carbon monoxide

|  | **Indicators Evaluated** |
| --- | --- |
| **Tumor Type** |  |
| Colorectal |  |
|  | 5-year overall survival |
|  | Adequate volume of resections |
|  | Adjusted 90-day mortality |
|  | All-cause 30-day mortality or any serious morbidity (DSM) |
|  | Carcinoembryonic antigen (CEA) obtained |
|  | Clinical staging performed |
|  | CM-V&O (combined measure of volume & outcome) |
|  | Failure-to-rescue rate (Percentage of patients with a severe complication who die) |
|  | Length of stay |
|  | Median number of days between first colonoscopy and first resection |
|  | Minimally invasive surgery (MIS) rate for colon cancer |
|  | MTL22 - (a) mortality and/or (b) transfer to another hospital (other than rehabilitation clinic) within 22 days after the index operation, and/or (c) postoperative length of hospital stay≥22 days. |
|  | MTL30 - (a) mortality and/or (b) transfer to another hospital (other than rehabilitation clinic) within 30 days after the index operation, and/or (c) postoperative length of hospital stay≥30 days. |
|  | Percentage achieving negative circumferential resection margins |
|  | Percentage of patients with a rectal cancer that underwent resection of the primary tumor |
|  | Proportion of colorectal patients who have pre-operative chest X-ray and abdominal ultrasound, CT scan or MRI |
|  | Proportion of colorectal patients who have undergone surgical resection for colon or rectal cancer have documentation that colonoscopy or barium enema with sigmoidoscopy was offered within 6 months before or after surgery |
|  | Proportion of patients who are diagnosed with colon cancer and do not have metastatic disease were offered a curative resection within 6 weeks of diagnosis. (Separating patients with diagnosis and treatment of care in the same hospital vs multiple sites) |
|  | Proportion of patients who are diagnosed with rectal cancer that appears clinically to be Stage II or III, and was offered surgical resections within 16 weeks after beginning of concurrent chemoradiotherapy (CCRT) |
|  | Proportion of stage I to III colorectal patients who undergo a wide surgical resection that have documented to be “negative margins” |
|  | Rate of adequate lymph node yield/examination |
|  | Rate of all-cause mortality within 30 days following surgery among patients with one or more complications (odds ratio) |
|  | Rate of anastomotic leakage (AL) |
|  | Rate of preoperative ostomy site marking |
|  | Rate of severe complications (Complication leading to a surgical, endoscopic, or radiological reintervention or to an in-hospital stay of more than 14 days, or to death) |
|  | Readmission within 30 days |
|  | Reoperation within 30 days |
|  | Risk-adjusted margin positivity rate (RAMP) |
|  | Sphincter-preserving surgery rate |
|  | Surgeon-reported Total Mesorectal Excision performance |
|  | Treatment within 60 days of diagnosis |
| Breast |  |
|  | Rate of breast conserving surgery (BCS) |
|  | Core needle biopsy rate |
|  | Irradical BCS for ductal carcinoma in situ (DCIS) |
|  | Irradical BCS for invasive disease |
|  | Length of stay |
|  | Percentage having surgery within 30 days of the first surgical consult |
|  | Percentage of Complete Axillary lymph node Dissection for positive sentinel lymph node biopsy (SLNB) |
|  | Percentage of node negative patients who underwent axillary dissection |
|  | Percentage of re-resections following BCS |
|  | Percentage of women with 'unknown' margin status |
|  | Proportion of axillary staging by SLNB only in pN0 |
|  | Proportion of DCIS patients receiving no axillary dissection |
|  | Proportion of patients having only one operation after pre-operative diagnosis |
|  | Proportion of patients undergoing mammogram within 14 months of definitive surgery for breast conservation or unilateral mastectomy |
|  | Proportion of patients undergoing reexcision of positive margins |
|  | Proportion of patients with positive margins |
|  | Proportion of surgeries for cancers <10mm where frozen section is NOT performed |
|  | Proportion of waiting times for surgery from first diagnostic test <42 days |
|  | Proportion of waiting times for surgery from referral ≤30 days |
|  | Proportion of waiting times for surgery from screening test <60 days and <90 days |
|  | Rate of ipsilateral breast tumor recurrence (IBTR) |
|  | Rate of SLNB |
|  | Surgery within 5 weeks of diagnosis. |
| Lung |  |
|  | 30-day mortality |
|  | 30-day or in hospital mortality rate |
|  | 90-day mortality |
|  | Average cost of care |
|  | Complication rate |
|  | Failure-to-rescue rate |
|  | In-hospital mortality |
|  | Length of stay |
|  | Odds of surgical resection for early small cell lung cancer |
|  | Percentage of regional lymph node evaluation |
|  | Proportion of cII–III non-small cell lung cancer (NSCLC) patients who had minimally invasive mediastinal staging (EBUS or EUS or mediastinoscopy) before treatment with curative intent |
|  | Proportion of NSCLC patients who died within 60 days after primary surgery |
|  | Proportion of NSCLC patients who had FEV1 and DLCO performed before surgery |
|  | Proportion of patients discussed in MDT within 6 weeks after incidence date |
|  | Rate of accuracy between cN and pN status |
|  | Rate of anatomic resection for T1b or greater |
|  | Rate of harvesting 10+ lymph nodes |
|  | Rate of lymph node sampling at the time of resection |
|  | Rate of major complications |
|  | Rate of R0 resection (negative margins) |
|  | Rate of sampling 5+ lymph node stations |
|  | Risk-adjusted margin positivity rate (RAMP) |
| Pancreas |  |
|  | 5-year survival rate after diagnosis |
|  | 90-day postoperative mortality |
|  | Rate of receipt of curative-intent surgery |
|  | Risk-adjusted margin positivity (RAMP) |
|  | Textbook outcome rate |
| Esophageal |  |
|  | 30-day mortality |
|  | 90-day mortality |
|  | Failure to cure |
|  | Proportion of adequate lymphadenectomies |
| Head & Neck | |
|  | Percentage of cases with adequate lymph node yield (LNY) = 18+ |
|  | Percentage of patients achieving negative margins |
|  | Proportion of patients receiving total thyroidectomy for papillary cancer>1.5 cm or node positive |
|  | Proportion of patients undergoing central neck dissection for node-positive papillary thyroid cancer |
|  | Proportion of patients undergoing lymph node dissection (LND) synchronously to first thyroid surgery |
|  | Proportion of papillary thyroid cancer tumors <4cm resected via total thyroidectomy |
|  | Rate of fine needle aspiration of thyroid cancers |
| Prostate |  |
|  | Adequate follow-up (2+ follow-up office visits within 1 year of treatment completion) |
|  | Incidence of repeat biopsy |
|  | Practice employs at least one board-certified urologist |
|  | Pretherapy functional assessment completed |
|  | Proportion of all biopsies performed for men with limited life expectancy |
|  | Psychological counseling available |
|  | Treatment options and risks discussed |
| Gyn |  |
|  | 30-day mortality |
|  | Proportion of resections performed using minimally-invasive surgery (MIS) |
|  | Complicated course rate |
|  | Textbook outcome rate |
| Gastric |  |
|  | Proportion of patients receiving adequate lymphadenectomy (AL) = examination of >15 nodes |
| Upper GI Tract | |
|  | 30-day complication rate |
|  | Composite of death or at least one of the following serious morbidities (DSM) occurring within 30 days |
|  | Patient reported experience |
| Renal |  |
|  | 30-day unplanned readmission proportion after radical nephrectomy for T1-4 tumors |
|  | Length of stay following radical nephrectomy |
|  | Proportion of cases for which 1) T1a tumors underwent partial nephrectomy and 2) T1-T2 tumors underwent minimally invasive radical nephrectomy |
|  | Proportion of positive surgical margins following partial nephrectomy |
|  | Proportion of T1a tumors undergoing partial nephrectomy. |
|  | Rate of minimally invasive surgery |
| Skin |  |
|  | Average number of follow-up visits per patient |
|  | Average time from initial excision to definitive surgery |
|  | Insufficient surgical margins |
|  | Proportion of patients treated via conventional excision versus Mohs micrographic surgery (MMS) |
|  | Proportion of SLNBs performed |
| Multiple tumor types in one evaluation | |
|  | 30-day mortality rate |
|  | 30-day readmission rate |
|  | 30-day serious morbidity rate |
|  | 90-day mortality rate |
|  | 90-day readmission rate |
|  | Availability and use of minimally invasive surgery |
|  | Average total episode payment (for 3 procedures) |
|  | Inability to receive planned chemotherapy |
|  | Involvement in research |
|  | Maintenance of a local thoracic surgery database or participate in a national or international database |
|  | Monitor morbidity and mortality regularly |
|  | Occurrence of Agency for Healthcare Research and Quality (AHRQ) patient safety indicators (PSIs) -- secondary infections, postoperative DVT/PE, wound dehiscence |
|  | Prolonged length of stay -- defined as admission duration longer than the 75th percentile (11 days after radical cystectomy, 5 days after nephrectomy, 3 days after prostatectomy) |
|  | Proportion of patients experiencing death or serious morbidity (DSM) |
|  | Proportion of patients provided uniformly guideline-concordant care |
|  | Risk-adjusted in-hospital mortality |
|  | Unplanned reoperation rate |

# **Supplemental Table 4. Quality Indicators by Tumor Type**

Abbreviations: cN = clinical nodal stage, pN = pathologic nodal stage, DVT = deep vein thrombosis, PE = pulmonary embolism, FEV1 = forced expiratory volume in 1 second, DLCO = diffusing capacity of lungs for carbon monoxide

## **Supplemental Table 5. Study details by tumor type**

*Colorectal*

| **Study; Year (Country)** | **Data Source; period** | **Hospitals evaluated; (patients evaluated)** | **Cancer type(s); stage** | **Quality Indicator(s)** | **Domain** | **Categorization, identification of outliers** | **Key Findings** | **Feedback to studied groups?** |
| --- | --- | --- | --- | --- | --- | --- | --- | --- |
| Adam; 2015 (USA)^14^ | National Cancer Database (NCD); 2004-2012 | 1,168 hospitals; (n = 185,464) | Colorectal; stage I-III | 30-day mortality  90-day mortality | Outcomes/  Adverse events  Outcomes/  Adverse events | Hospitals were ranked into top (10%), middle (80%), and lowest (10%) performance groups.  Risk-adjusted: Yes | 90-day mortality was nearly double the 30-day mortality (4.4% vs. 2.5%).  Following risk adjustment 176 hospitals changed performance ranking: 39% in the top 30-day mortality group changed ranking to the middle group; 37% of hospitals in the lowest 30-day group changed ranking to the middle 90-day group. | No |
| Hardt; 2019 (Germany)^15^ | German Society of General and Visceral Surgery (DGAV) national database; 2010-2017 | 144; (n = 14,987) | Colorectal; Stage I-IV | MTL22 - (a) mortality and/or (b) transfer to another hospital (other than rehabilitation clinic) within 22 days after the index operation, and/or (c) postoperative length of hospital stay≥22 days.  MTL30 (same as above, but 30days) | Outcomes/  Adverse events  Outcomes/  Adverse events | Compared to one another via funnel plot  Risk-adjusted: No | For MTL22, 10 hospitals (7%) were located above the upper 99.8% control limits and 17 hospitals (12%) lay above the upper 95% control limits compared to 10 hospitals (7%) exceeding the upper 99.8% control limits and 14 hospitals (10%) the upper 95% control limits for MTL30.  Seven hospitals (5%) were located below the lower  99.8% control limits and 12 hospitals (8%) below the lower  95% control limits for MTL22, whereas, regarding MTL30, 2 hospitals (1%) lay below the lower 99.8% control limits and 10 hospitals (7%) below the lower 95% control limits. | No |
| Antunez; 2019 (USA)^16^ | NCD; 2011-2015 | 1,315 hospitals; (n = unreported) | Rectal; stage I-IV | Clinical staging performed  Carcinoembryonic antigen (CEA) obtained  Treatment within 60 days of diagnosis | Appropriateness  Appropriateness  Timeliness | Sorted into 4 cohorts, organized by high vs low volume and adherence to process standards, and patient and hospital characteristics and oncologic outcomes compared  Risk-adjusted: No | 38 (2.9%) met proposed thresholds for all 5 process standards and 220 (16.7%) met the threshold on 4 standards. | No |
| Abdelsattar; 2014 (USA)^17^ | Michigan Surgical Quality Collaborative (MSQC); 2008-2012 | 52 hospitals; (n = 199,990 [w/cancer = 7,364) | Colorectal; stage I-IV | 30-day morbidity rate  Risk-adjusted major morbidity rate  Extended length of stay rate (LOS>75th percentile)  30-day mortality | Outcomes/  Adverse events  Outcomes/  Adverse events  HC delivery &  Outcomes/  Adverse events  Outcomes/  Adverse events | Compared to one another by O:E ratio  Risk-adjusted: Yes | Wide variations in all risk-adjusted 30-day outcomes between hospitals, but only weak correlations in  cancer and noncancer performance within hospitals. | No |
| Kanters; 2017 (USA)^18^ | MSQC; 2014-2015 | 30 hospitals; (n = 871) | Colorectal; stage I-IV | Mesorectal excision rate  Rate of minimally invasive surgery (MIS) for colon cancer  Rate of sphincter preservation surgery (SPS) for rectal cancer  Rate of adequate lymph node yield/examination  Margin positivity rate  Rate of preoperative ostomy site marking (by nurse) | Appropriateness & Technical  Technical  Technical  Appropriateness  Technical  Multidisciplinary | Compared to one another  Risk-adjusted: Yes | We find significant variation in use of mesorectal excision for rectal cancer. While incomplete documentation may be to blame, hospitals' rates vary from less than 20% to more than 90% of cases, suggesting true variation in the importance placed on mesorectal excision between hospitals.  Significant variability in the use of MIS surgery, ranging by hospital from less than 20% of cases to more than 70%.  Due to small numbers, there were not statistically significant outlier hospitals for rates of SPS, but crude rates seem to vary by hospital (data not shown) | Yes  Fed into the Michigan Surgical Quality Collaborative. |
| Lucas; 2014 (USA)^19^ | Surveillance, Epidemiology, and End Results (SEER) database; 1997-2022 | 1,401 hospitals; (n = 44, 822) | Colorectal; stage I-IV | 30-day readmission rate | Outcomes/  Adverse events | Compared to one another via O:E ratio  Risk-adjusted: Yes | Found marked variation in raw readmission rates, with a range of 0% to 41.2% (IQR, 9.5%-14.8%). However, after adjusted for patient characteristics, comorbidities, and operation types in a hierarchical model, no significant variability was found in readmission rates--range of 11.3% to 13.2% (IQR, 12.1%-12.4%). 95% CI for hospital-specific readmission overlapped the overall mean at every hospital. | No |
| Merkow; 2009 (USA)^20^ | National Surgical Quality Improvement Program (NSQIP); 2006-2007 | 182 hospitals; (n = 23,098) | Colorectal; stage I-IV | Rate of reoperation w/in 30 days of index surgery | Outcomes/  Adverse events | Compared to one another via O:E ratio  Risk-adjusted: Yes | Reoperation occurred significantly more often than expected in 16 hospitals and less often than expected in 7 hospitals (p < 0.05). | No |
| Merkow; 2013 (USA)^21^ | NSQIP; 2010 | 255 hospitals; (n = 22,346) | Colorectal; stage I-IV | Rate of all-cause 30-day mortality or any serious morbidity | Outcomes/  Adverse events | Compared to one another via O:E ratio  Risk-adjusted: Yes | At the hospital level, the median composite event rate was 20.7% (interquartile range:  15.8%–26.3%).  The full model identified  3 high outliers and 7 low outliers, and the parsimonious model identified 3 high outliers and 8 low outliers. | No |
| Kanters; 2020 (USA) ^22^ | MSQC; 2007-2016 | 38 hospitals; (n = 510) | Colorectal; stage I-IV | Rate of TME performance (abstracted from operative reports) | Appropriateness & Technical | Compared to one another  Risk-adjusted: No | Between-hospital variability in total mesorectal excision performance ranged from 0% to 97% | Yes  Fed into QI efforts at the regional level, including designing an educational intervention to encourage TME-grading. |
| McSorley; 2013 (UK) ^23^ | Administrative & regional clinical data registry; 2006-2008 | 3 hospitals; (n = 537) | Colorectal; stage I-IV | Rate of reoperation within 30 days  Average length of stay | Outcomes/  Adverse events  HC delivery & Outcomes/  Adverse events | Compared to one another  Risk-adjusted: No | Average unplanned rate of reoperation within 30 days was 5.4%. There was no statistically significant difference between hospital sites. | No |
| Morris; 2008 (UK) ^24^ | Health Episode Statistics (HES) & regional cancer registries; 1998-2004 | 153 hospitals; (n = 31,223) | Rectal; stage I-IV | Rate of abdominoperineal excision (APE) | Appropriateness & Technical | Compared to one another via odds ratio  Risk-adjusted: Yes | Significant variations in the odds of receiving an APE between hospital trusts independent of patient case-mix. | No |
| Morris; 2011 (UK) ^25^ | National Cancer Data Repository (NCDR); 1998-2006 | 150 hospitals; (n = 160,920) | Colorectal; stage I-IV | 30-day postoperative mortality | Outcomes/  Adverse events | Compared to one another using funnel plot  Risk-adjusted: Yes | Risk-adjusted control charts showed that one trust had consistently significantly better outcomes and three had significantly worse outcomes than the population mean. | Yes  State their intention to publicly report these data and report at surgeon-level in coming years |
| Giesen; 2021 (Netherlands) ^26^ | Netherlands Cancer Registry; 2013-2018 | ? hospitals; (n = 22,640) | Rectal; stage I-IV | Percentage that underwent resection of the primary tumor | Appropriateness | Compared to one another directly and via odds ratio for quartiles  Risk-adjusted: Yes | Resection rates varied from 68 to 89% between hospitals. After multivariable analysis, resection rate remained significantly different among quartiles when correcting for several factors (odds ratio (95%Confidence-interval) 1.71 (1.56-1.88), 2.42 (2.19-2.67), and 4.04 (3.61-4.53) for increasing resection rate quartiles, in reference to the lowest quartile). | Yes  Fed into QI efforts at the national level. |
| Almoudaris; 2013 (UK) ^27^ | HES; 2000-2008 | 149 hospitals; (n = 144,542) | Colorectal; stage I-IV | Rate of extended LOS  30-day readmission rate  30-day mortality  Rate of reoperation  Rate of APE  Failure-to-rescue (FTR) surgical rate | HC delivery & Outcomes/  Adverse events  Outcomes/  Adverse events  Outcomes/  Adverse events  Outcomes/  Adverse events  Appropriateness & Technical  Outcomes/  Adverse events | Compared to one another via funnel plot  Risk-adjusted: Yes | 5 high mortality outlier (HMO) units and 15 low mortality outlier (LMO) units were identified. Of the 5 HMO units, two were substandard performance outliers (>3 SD) on high reoperation rates. A further 2 HMO institutions exceeded the second but not the third SD limits for substandard performance on other outcome metrics. One of the 15 LMO units exceeded 3 SD for substandard performance on APE rate. One LMO institution exceeded the second but not the third SD control limits for high reoperation rates. | No |
| Massarweh; 2014 (USA) ^28^ | NCD; 2003-2005 | 1,349 hospitals; (n =  32,354) | Colorectal; stage I-III | 5-year overall survival  Risk-adjusted margin positivity rate (RAMP)  Proportion of patients with adequate lymph node evaluation  Rate of SPS | Outcomes/  Adverse events  Technical  Appropriateness  Technical | Compared to one another via O:E ratio  Risk-adjusted: Yes | 4.9% of hospitals were high RAMP outliers, 0.7% were low outliers. 5.6% of patients were treated at high outliers and 3.0% at low outliers. | No |
| Abdelsattar; 2014 (USA) ^29^ | MSQC & medical records; 2007-2012 | 52 hospitals; (n = 329) | Rectal; stage I-IV | Rate of SPS | Technical | Compared to one another via O:E ratio  Risk-adjusted: Yes | Crude SPS rates varied by hospital, with a mean rate of 72% (range = 47-91%). Hospitals varied significantly in their SPS rates, even with adjustment for clinical characteristics. | Yes  Fed into regional QI efforts. |
| Chung; 2010 (Taiwan) ^30^ | Taiwan Cancer Database; 2004 | 11 hospitals; (n = 5,585) | Colorectal; stage I-IV | Proportion with pre-operative chest X-ray and abdominal ultrasound, CT scan or MRI.  Proportion with documented colonoscopy or barium enema w sigmoidoscopy offered within 6mos before or after surgery  Proportion offered curative resection w/in 6 weeks  Proportion with wide surgical resection and documented “negative margins” (stage I-III)  Proportion with 12+ lymph nodes evaluated (stage I-III)  Proportion offered resection w/in 16 weeks of beginning CCRT (stage II-III) | Appropriateness  Appropriateness  Timeliness  Technical  Appropriateness  Appropriateness & Timeliness | Compared to one another using mean as benchmark  Risk-adjusted: Yes | 2 indicators had mean adherence less than 70%. The best and poorest system performance occurred for negative surgical margin (99.3%, ranged from 97.2% to 100.0%) and 12+ lymph nodes (62.7%, ranged from 27.6% to 92.2%). | Yes  Used to inform development of core measures for cancer care in Taiwan. |
| Henneman; 2013 (Netherlands) ^31^ | Dutch Surgical Colorectal Audit; 2009-2011 | 92 hospitals; (n = 24,667) | Colorectal; stage I-IV | 30-day mortality rate  Rate of severe complications  Failure-to-rescue (FTR) rate | Outcomes/  Adverse events  Outcomes/  Adverse events  Outcomes/  Adverse events | Compared to one another via funnel plot  Risk-adjusted: Yes | Severe complications ranged from 19 % in the lowest to 25 % in the highest mortality quintile (odds ratio 1.5, 95 % confidence interval 1.37–1.67).  7 hospitals had significantly lower FTR rates than average | Yes  Sent results to all studied. |
| Schootman; 2014 (USA) ^32^ | Billing data & SEER database; 1999-2005 | 1,222 hospitals; (n =  35,946) | Colorectal; stage I-III | Rate of postoperative complications  Rate of all-cause 30-day mortality among patients w complication(s) | Outcomes/  Adverse events  Outcomes/  Adverse events | Compared to one another via median odds ratio  Risk-adjusted: Yes | Hospital variability in death after complications (0.16) was two times larger than hospital variability in incidence of complications (0.08) | No |
| Ratnayake; 2021 (Canada) ^33^ | Admin, billing, & regional data registry; 2010-2014 | 5 hospitals; (n = 3,445) | Colorectal; stage I-IV | Proportion of resections done laparoscopically  Median days b/n first colonoscopy and resection  Proportion of resections w 12+ LNs removed  % w negative circumferential resection margins | Technical  Timeliness  Appropriateness  Technical | Compared to one another via mean  Risk-adjusted: Yes | For colon cancer, the percentage of laparoscopic surgery by RHA ranged from 12.9% to 29.2%. Laparoscopic surgery for rectal cancer showed less variation, with a range of 13.2% to 18.1% by RHA  Little variation was identified between RHAs, and all stages showed a high percentage of lymph node removal (74.8% to 90.9%). The negative circumferential resection margin rate for rectal cancers was 96.9%, ranging between 96.0% and 100.0% by RHA of residence | Yes  A formal structure for ongoing measuring and reporting surgical quality has been established in Manitoba. Quality improvement initiatives have been implemented based on these findings and periodic assessments of colorectal cancer surgery quality will continue. |
| Patel; 2020 (USA) ^34^ | NCD; 2010-2015 | 1,303 hospitals; (n = 58,374) | Rectal; stage I-III | Rate of circumferential resection margin positivity | Technical | Compared to one another via O:E ratio  Risk-adjusted: Yes | 147 (12.9%) and 103 (9.0%) hospitals were significantly worse and better performers, respectively. | Yes  Fed into QI efforts at the national level, facility-specific tool developed to allow access to these results. |
| Snijders; 2013 (Netherlands) ^35^ | Dutch Surgical Colorectal Audit; 2009-2011 | 92 hospitals; (n = 25,555) | Colorectal; stage I-IV | Rate of anastomotic leakage  30-day mortality rate | Outcomes/  Adverse events  Outcomes/  Adverse events | Compared to one another via O:E ratio  Risk-adjusted: Yes | Variation in observed AL rates between hospitals was large with a maximum rate of 17%.  Hospital variation in observed mortality rates was significantly reduced after adjustment for differences in case-mix | No |
| Kolfschoten; 2014 (Netherlands) ^36^ | Dutch Surgical Colorectal Audit; 2010-2011 | 89 hospitals; (n = 8,911) | Colorectal; stage I-IV | Mortality rate  Rate of serious morbidity  Combined measure of volume and outcomes (CMV&O) | Outcomes/  Adverse events  Outcomes/  Adverse events  HC delivery & Outcomes/  Adverse events | Compared to one another via O:E ratio  Risk-adjusted: Yes | 84 (94%) hospitals performed ‘not worse than average’ for mortality, but only 21 (24%) of those were able to prove they were also ‘better than substandard’ (O/E,2). For morbidity, 42 hospitals (47%) met the CM-V&O. | Yes  Fed into clinical audit, results shared with participants. |
| Bertoy; 2023 (USA) ^103^ | Michigan Surgical Quality Collaborative (MSQC) database; 2016-2020 | 42 hospitals; (n = 4,211) | Colorectal; stage I-IV | Margin positivity following primary CRC resection | Technical | Compared to one another via O:E ratio  Risk-adjusted: Yes | The crude positive margin rate was 6.15 % (95 % CI 4.6–7.4 %); this ranged from 0 % to 22 % at individual hospitals. After risk-adjustment, there remained significant variation by hospital, with 8 hospitals being statistically significant outliers. | Yes  Fed into regional quality improvement efforts, results shared with participants. |
| Xu; 2023 (New Zealand) ^104^ | New Zealand Ministry of Health National Minimum Dataset (NMDS) and the New Zealand Cancer Registry (NZCR); 2010-2020 | 26 hospitals; (n = 16,885) | Colorectal; stage I-IV | 90-day readmission rate  30-day readmission rate | Outcomes/adverse events  Outcomes/adverse events | Compared to one another via O:E ratio  Risk-adjusted: Yes | Unplanned 30-day and 90-day hospital readmission rates were 15.1% and 23.7% respectively. Hospital-level variation was not present. | No |
| Manisundaram; 2023 (USA) | National Cancer Database  (NCD); 2010-2019 | 1307 hospitals; (n = 110,571) | Rectal; stage I-III | Rate of Circumferential Resection Margin (CRM) status ‘missingness’ | Clinical practice guideline adherence | Compared to one another with benchmark  Risk-adjusted: Yes | Reporting of CRM improved over the study period, with a mean (SE) missing 12.0% (0.32%) decreased from 16.3% (0.36%). Academic facilities had a higher missingness than other facility types (14.3% vs 10.5%-12.7%; P < .001). Mean (SE) rates of missingness were similar between hospitals of varying volume (lowest quartile: 12.2% [0.93%] vs highest quartile: 12.4% [0.53%]; P = .96). Cases in which fewer than 12 lymph nodes were removed had higher rates of missingness (18.1% vs 11.4%; P < .001). | No |

*Breast*

| **Study; Year (Country)** | **Data Source; period** | **Hospitals evaluated; (patients evaluated)** | **Cancer type(s); stage** | **Quality Indicator(s)** | **Domain** | **Categorization, identification of outliers** | **Key Findings** | **Feedback to studied groups?** |
| --- | --- | --- | --- | --- | --- | --- | --- | --- |
| Breslin; 2011 (USA)^37^ | Michigan Breast Oncology Quality Initiative (MiBOQI) & National Comprehensive Cancer Network (NCCN) Oncology Outcomes Database Project; 2006-2009 | 14 hospitals; (n = 8,066) | Breast; Stage I-III | Surgical biopsy rate for initial diagnosis | Appropriateness | Hospitals compared according to pre-determined benchmark.  Risk-adjusted: No | Collaborative members identified target of <15% surgical biopsies.  Only two hospitals were positive outliers initially.  Over the study period, the percentage of cases undergoing surgical biopsy for the collaborative decreased from 21% to 15% (P<.001). | Yes  Center-specific benchmarked data provided to project directors quarterly. Aggregate, blinded data and patterns shared at regional meetings. |
| Gort; 2007 (Netherlands)^38^ | Regional clinical registry data & medical records; 1998-2022 | 16 hospitals; (n = 2,929) | Breast; Stage I-II | Breast conserving surgery (BCS) rate | Appropriateness | Individual centers compared to one another and mean  Risk-adjusted: Yes | The mean BCS rate for the hospitals was 51.1% (SD 13.1). The proportion of patients treated with BCS differed significantly between hospitals, varying from 25.5% to 79.3% for cT1 tumors and from 16.7% to 48.1% for cT2 tumors | No |
| Boughey; 2014 (USA)^39^ | NSQIP & administrative data; 2006-2010 | 1 hospital; (n = 24,217) | Breast; Stage I-IV | Reoperation rate | Outcomes/  Adverse events | Single center compared to national average  Risk-adjusted: No | The 30-day reoperation rate after lumpectomy was greater nationally than at the FS institution (13.2% vs 3.6%, P < .001) | No |
| Laronga; 2014 (USA)^40^ | Medical records; 2006-2009 | 11 hospitals; (n = 1,242) | Breast; Stage I-IV | Sentinel lymph node biopsy (SNLB) rate  Complete axillary lymph node dissection (CALND) rate for positive SLNB  Proportion of patients undergoing mammogram within 14 months of definitive surgery for breast conservation or unilateral mastectomy | Appropriateness  Appropriateness  Appropriateness | Individual centers compared to one another graphically  Risk-adjusted: Yes | In 2006, sentinel lymph node biopsy (SLNB) was performed in 82% of all patients with invasive breast cancer; in 2009, adherence increased to 87% (p = 0.035).  Of the patients with a metastatic SLNB, 79% of patients in 2006 and 86% of patients in 2009 went on to have a complete axillary node dissection (CALND) (p = 0.104).  Obtaining a mammogram within 14 months of definitive surgery for breast conservation or unilateral mastectomy patients was not significantly improved, with adherence of 77% and 79% in 2006 and 2009, respectively (p = 0.381). | Yes  Fed into quality improvement efforts at the state level |
| Ponti; 2015 (Italy)^41^ | National clinical data registry (Audit System on Quality of Breast Cancer Treatment [QT]); 2011-2012 | 8 Regions (n = 8,809) | Breast; Stage I-IV | Proportion of waiting times for surgery from referral ≤30 days (40, 60, and 90 days)  Proportion of surgeries for cancers <10mm where frozen section is NOT performed  Proportion of patients having only one operation after pre-operative diagnosis  Proportion of patients undergoing conservative surgery in invasive cancers or DCIS  Proportion of axillary staging by SLN only in pN0  Proportion of DCIS patients receiving no axillary dissection | Timeliness  Appropriateness  Outcomes/  Adverse events  Appropriateness  Technical  Appropriateness | Regions compared to pre-determined benchmark  Risk-adjusted: No | Wait times varied significantly by region.  The proportion of node-negative invasive cases staged by SLN biopsy only was 91%, with moderate variability by region (range 73%-100%).  3.3% of DCIS (range between regions: 0%- 7%) received clearance of the axilla. | Yes  Results distributed to regional and local screening programs, to allow multidisciplinary discussion and identification of the appropriate solutions to any issues documented by the data. |
| deMunck; 2012 (Netherlands)^42^ | Netherlands Cancer Registry; 2008-2009 | 96 hospitals; (n = 7,345) | Breast; stage I-II | Percentage of women with 'unknown' margin status  Proportion of patients with positive margins | Technical  Technical | Compared to one another (funnel plot) and pre-determined benchmark  Risk-adjusted: Yes | Overall, 28.5% (95% CI: 25.5–31.4%) of DCIS and 9.1% (95% CI: 8.4–9.8%) of invasive carcinoma had positive margins. Variation between hospitals was substantial. After case mix correction, 10 hospitals showed significantly higher rates than above limit of agreement, while 7 hospitals showed significantly lower rates. | No |
| McCahill; 2012 (USA)^43^ | Medical records; 2003-2008 | 4 hospitals; (n = 2,206) | Breast; stage I-IV | Proportion of patients undergoing reexcision of initial negative margins  Proportion of patients undergoing reexcision of positive margins | Technical  Technical | Compared to one another  Risk-adjusted: Yes | For patients with negative margins, reexcision rates  varied widely among surgeons (range, 0%-70%; P=.003) and institutions (range, 1.7%-  20.9%; P.001) | No |
| Siesling; 2015 (Netherlands)^44^ | Netherlands Cancer Registry; 2003-2006 | 92 hospitals; (n = 40,892) | Breast; stage I-III | Rate of ipsilateral breast tumor recurrence (IBTR) | Outcomes/  Adverse events | Compared to one another (funnel plot) and pre-determined benchmark  Risk-adjusted: Yes | The 5-year IBTR rate was 2.85 % (95 % confidence interval 2.68–3.03) and was significantly lower for BCS than for mastectomy (2.38 vs. 3.45 %, p=0.001). IBTR rates decreased over time in both groups.  Rates varied between 0.77 and 5.70% between hospitals. When random variation is taken into account, only extremely high IBTR rates can be detected as deviant from the target value of 5 %. | No |
| Jeevan; 2012 (UK)^45^ | HES; 2005-2008 | 156 trusts; (n = 55,297) | Breast; stage I-II | Rate of reoperation after breast conserving surgery | Outcomes/  Adverse events | Compared to one another using funnel plot  Risk-adjusted: Yes | Substantial differences were found in the adjusted reoperation rates among the NHS trusts (10th and 90th centiles 12.2% and 30.2%). | No |
| Talsma; 2011 (Netherlands)^46^ | Rotterdam Cancer Registry (RCR); 2006-2007 | 16 hospitals; (n = 961) | Breast; stage I-II | Rate of re-resections following BCS | Outcomes/  Adverse events | Compared to reference hospital via VLAD chart  Risk-adjusted: Yes | One hospital was performing significantly less re-resections compared to the reference hospital and two were performing significantly more re-resections than was expected. |  |
| Vos; 2020 (Netherlands)^47^ | National Breast Cancer Working Group Netherlands (NABON) Breast Cancer Audit (NBCA); 2011-2016 | 91 hospitals; (76,690) | Breast; stage I-IV | Rate of irradical BCS for invasive disease  Rate of irradical BCS for DCIS  Rate of breast contour–preserving treatment  Proportion receiving surgery within 5 weeks of diagnosis. | Appropriateness  Appropriateness  Appropriateness  Timeliness | Compared to one another by O:E ratio  Risk-adjusted: Yes | All the indicators showed between-hospital variation with wide (interquartile) ranges. Case-mix adjustment  reduced variation in 3 indicators. | No |
| Gray; 2011 (USA)^48^ | Medical records; 2006-2009 | 11 hospitals; (n = 622) | Breast; stage I-IV | Percentage of SLNB in clinical N0 patients  Percentage of CALND for positive SLNB  Percentage obtaining a mammogram within 14 months of definitive surgery | Appropriateness  Appropriateness  Appropriateness | Compared to pre-determine benchmark  Risk-adjusted: Yes | A statistically significant difference (P < .001) in variation of performance across the sites was found for the surgical oncology indicators: having a sentinel lymph node biopsy for invasive BC, and obtaining a mammogram within 14 months of definitive surgery | Yes  All institutions were provided with the results and were un-blinded as to which letter corresponded to their particular site. These findings are now being used at the participating institutions to guide quality improvement efforts. |
| Ratnayake; 2021 (Canada) ^49^ | Admin, billing, and regional clinical data registry; 2010-2014 | 5 health authorities; (n = 3,962) | Breast; stage I-III | Percentage of node negative patients who underwent axillary dissection  Percentage having surgery within 30 days of first surgical consult  Percentage requiring re-excision after BCS | Appropriateness  Timeliness  Outcomes/  Adverse events | Compared to one another  Risk-adjusted: No | Axillary lymph node dissection (ALND) for node-negative disease ranged from 3.4% to 32.6%, timeliness (surgery within 30 days of consult) ranged from 33.3% to 60.2%, and re-excision ranged from 14.7% to 24.6% between health authorities. | No |
| Ishizaki; 2002 (Japan)^50^ | Voluntary Hospitals of Japan Quality Indicator Project; 1996-1999 | 5 hospitals; (n = 614) | Breast; stage I-IV | Proportion of BCS use among operable breast cancer patients  Length of stay | Appropriateness  HC delivery &  Outcomes/  Adverse events | Compared to one another  Risk-adjusted: Yes | Proportions of use of BCS varied from 9-51% across the 5 hospitals.  LOS also varied significantly. | No |

*Lung*

| **Study; Year (Country)** | **Data Source; period** | **Hospitals evaluated; (patients evaluated)** | **Cancer type(s); stage** | **Quality Indicator(s)** | **Domain** | **Categorization, identification of outliers** | **Key Findings** | **Feedback to studied groups?** |
| --- | --- | --- | --- | --- | --- | --- | --- | --- |
| Wakeam; 2017 (USA) ^51^ | NCD; 2004-2013 | 317 hospitals; (n = 1,260) | SCLC; stage I-II | Odds of surgical resection  Percentage undergoing regional lymph node evaluation | Appropriateness  Appropriateness | Compared to one another via O:E ratio  Risk-adjusted: Yes | Wide variation in the use of surgical management between the highest compared with the lowest quartiles of surgical management (44.8% vs 7.5%)  Patients treated at hospitals in the highest quartile were nearly 17 times more likely to receive surgical treatment (OR, 16.7; 95% CI, 12.59 to 22.18).  Significantly greater rates of lymph node evaluation were observed in hospitals with the highest rates of surgical management than in those in the lowest quartile for all patients (39.2% vs 7.4%, p < 0.001). | No |
| Hu; 2014 (USA) ^52^ | SEER database & billing data; 2006-2011 | 686 hospitals; (n = 11,787) | NSCLC; stage I-IV | In-hospital mortality rate  Perioperative mortality (in-hospital or w/in 30 days)  30-day mortality  90-day mortality | Outcomes/  Adverse events  Outcomes/  Adverse events  Outcomes/  Adverse events (x2) | Compared to one another via O:E ratio  Risk-adjusted: Yes | The 90-day mortality O/E ratio was >1 for 18 providers and <1 for 2 providers.  30-day O/E ratio >1 for 19, <1 for 1. | No |
| Geraci; 2017 (USA) ^53^ | Admin data; 2004-2013 | 2 hospitals; (n = 419) | NSCLC; stage I-IV | LOS  Rate of major complications  Failure-to-rescue rate  30-day mortality  90-day mortality | HC delivery &  Outcomes/  Adverse events  Outcomes/  Adverse events  Outcomes/  Adverse events  Outcomes/  Adverse events (x2) | Compared to each other after propensity score matching  Risk-adjusted: Yes | Higher rate of major complications (12% versus 27%, p = 0.02) and longer hospital stay (median 6.0 versus 7.5 days, p < 0.001) for VAMC, but no difference in 90-day mortality (AMC 5% versus VAMC 6%, p > 0.99) | No |
| Khorfan; 2021 (USA) ^54^ | NCD; 2010-2015 | 1,324 hospitals; (n = 253,182) | NSCLC; stage I-II | Rate of lymph node sampling at resection  Rate of anatomic resection for T1b+  R0 resection rate | Appropriateness  Appropriateness  Technical | Compared to predetermined benchmark  Risk-adjusted: Yes | Lymph node sampling was performed in 91% of patients nationally, but only 76% of hospitals met the 85% adherence mark. Similarly, 89% of T1b (seventh edition staging) tumors had anatomic resection, with 69% hospital-level adherence.  96% of hospitals achieved R0 resection rate >85% | No |
| Farjah; 2015 (USA) ^55^ | Society of Thoracic Surgeons General Thoracic Surgery Database; 2009-2012 | 208 hospitals; (n = 30,000) | Lung; stage I-III | Complication rate  Failure-to-rescue (FTR) rate | Outcomes/  Adverse events  Outcomes/  Adverse events | Compared to each other using mean  Risk-adjusted: Yes | Mortality rates varied over 4-fold across hospitals (3.2% vs 0.7%).  The magnitude of variation (22%) in complication rates dwarfed the 4-fold magnitude of variation in failure to rescue rates (6.8% vs 1.7%, P<0.001) across hospitals | No |
| TenBerge; 2018 (Netherlands) ^56^ | Dutch Society of Lung Surgeons (NVvL) and the Dutch Society of Cardiothoracic Surgeons (NVT) Audit; 2012-2015 | 43 hospitals; (n = 19,557) | NSCLC; stage I-IV | 30-day or in-hospital mortality rate | Outcomes/  Adverse events | Compared to each other via funnel plot  Risk-adjusted: Yes | The 30-day or in hospital mortality was low and varied between centers from 0-5.3% for lobectomies or segment resections and 0-22.8% for pneumonectomies (overall 30-day mortality: 1.5% to 2.9%) | Yes  Sent to all studied |
| Thornblade; 2018 (USA) ^57^ | Surgical Clinical Outcomes and Assessment Program in Washington State; 2011-2013 | 5 hospitals; (n = 406) | NSCLC; stage I-IV | Rate of invasive mediastinal staging (IMS) | Appropriateness | Compared to one another via O:E ratio  Risk-adjusted: Yes | 2 hospitals performed IMS significantly more often than the overall average (94%, [95% CI, 89%-96%] and 84% [95% CI, 78%-88%]), whereas 2 hospitals performed IMS significantly less often than overall average (31% [95% CI, 21%-44%] and 17% [95% CI, 7%-36%]). | No |
| Lin; 2017 (USA) ^58^ | NCD; 2004-2011 | 809 hospitals; (n = 96,324) | NSCLC; stage I-III | RAMP rate | Technical | Compared to one another via O:E ratio  Risk-adjusted: Yes | 61 hospitals (8%) were outperformers, 644 (80%) were nonoutliers, and 104 (13%) were underperformers. | No |
| Vrijens; 2018  (Belgium) ^59^ | Belgian Cancer Registry (BCR) & claims data via Intermutualistic Agency (IMA); 2010-2011 | ? hospitals; (n = 12,839) | Lung; stage I-IV | Proportion of patients discussed in MDT within 6 weeks after incidence date  Proportion with IMS before treatment (II-III NSCLC)  Proportion who had FEV1 and DLCO performed before surgery (NSCLC)  60-day mortality rate (NSCLC) | Multidisciplinary  Appropriateness  Appropriateness  Outcomes/  Adverse events | Compared to one another via funnel plot  Risk-adjusted: Yes | High variability between centres was observed for several indicators. (details in Vrijens’ supplement) | Yes  Sent to all studied. |
| Farjah; 2014 (USA) ^60^ | Washington State Discharge Database; 2000-2011 | 24 hospitals; (n = 7,693) | stage not specified | Inpatient death rate  Prolonged length of stay  LOS | Outcomes/  Adverse events  HC delivery &  Outcomes/  Adverse events  HC delivery & Outcomes/  Adverse events | Compared to one another using mean as benchmark  Risk-adjusted: Yes | 5 hospitals were statistical outliers in rates of death (4 lower and 1 higher than the state average), and 13 were outliers with respect to prolonged length of stay (7 higher and 6 lower than the state average) and costs (5 higher and 8 lower than the state average) | No |
| Udelsman; 2021 (USA) ^61^ | Society of Thoracic Surgeons General Thoracic Surgery Database; 2012-2017 | 194 hospitals; (n = 39,516) | Lung; stage I-III | Rate of accuracy between cN and pN status | Technical | Compared to one another by mean  Risk-adjusted: Yes | The accuracy of the top centers was 94.4%, whereas in the bottom centers it was 70.9%. Low-performing centers had a 6 times greater rate of overstaging than top centers (6.5 vs 1.0, P < .001) | No |
| Moore; 2019 (USA) ^62^ | NCD; 2004-2013 | 1,190 hospitals; (n = 303,579) | NSCLC; stage I-IV | 30-day mortality  90-day mortality | Outcomes/  Adverse events (x2) | Compared to one another by odds ratio  Risk-adjusted: Yes | Eleven out of 30 facilities were in the top 2.5% at 30 days and moved to the middle 95% at 90 days  12 out of 30 facilities that were in the bottom ranking group at 30 days moved up into the middle ranking group at 90 days | No |
| Ivanovic; 2011  (Canada) ^63^ | Center-reported data; 2009 | 28 hospitals; (n =NA) | Thoracic cancer (all types); stage NA | Involvement in research  Maintenance of or participation in thoracic surgery database  Monitor morbidity and mortality regularly  Availability and use of MIS | HC delivery &  Innovation  HC delivery &  Innovation  Appropriateness  Technical | Compared to one another based on typical response  Risk-adjusted: No | 21 institutions (75.0%) are directly involved in research and 10 (38.5%) have a local thoracic surgery database. Most institutions do not participate in a national or international thoracic surgery database (92.3%; n = 24).    Most institutions (73.1%; n = 19) do not have a formal surgery quality assessment program but do however monitor M&M regularly (69.2%; n = 18).  Minimally invasive thoracic surgery is available and used at 25 institutions (96.2%). Survey results reveal that up to 50% of lobectomies are performed via video-assisted thoracoscopic surgery (73.1%; n = 19 institutions). | No |
| Bernard; 2023 (France) | Programme de Médicalisation des Systèmes d’Information; 2013-2020 | 199 hospitals; (n = 87,232) | Lung cancer (all types); stage NA | 30-day mortality | Outcomes/adverse events | Compared to one another using O:E ratio  Risk-adjusted: Yes | Among the regions that had the most hospitals performing lung resections for cancer, the extreme ratio was >2, which means that the maximum value is twice as high as the minimum value. The SCV between hospitals was >10 for two of these regions, which is considered indicative of very high variation. For the other regions (with few hospitals performing lung resections for cancer), the variation between hospitals was lower. Globally, the variability between regions concerning the SMR was moderate, 6% of the variance was due to differences across regions. On the contrary, the hospital volume was significantly related to the SMR (p = 0.003) with a negative linear trend, whatever the region. | No |
| Kalata; 2023 (USA) | Michigan Society of Thoracic and Cardiovascular Surgeons Quality Collaborative & Society of Thoracic Surgeons General Thoracic Surgery Database; 2015-2020 | 16 hospitals; (n = 3,753) | NSCLC; stage IA-IIIA | Rate of harvesting 10+ lymph nodes  Rate of sampling 5+ lymph node stations | Appropriateness of care/technical aspects  Appropriateness of care/technical aspects | Compared to guideline benchmark  Risk-adjusted: No | There is wide variation in adherence to each individual quality metric by the participating institutions.  The rates of examining 10 lymph nodes or more statewide increased from 215 lobectomies (44.0%) in 2015 to 522 lobectomies (78.9%) in 2020. | Yes  Quality improvement activities at biannual meetings, surgeon champion work at hospitals, iterative feedbacl |

*Pancreatic*

| **Study; Year (Country)** | **Data Source; period** | **Hospitals evaluated; (patients evaluated)** | **Cancer type(s); stage** | **Quality Indicator(s)** | **Domain** | **Categorization, identification of outliers** | **Key Findings** | **Feedback to studied groups?** |
| --- | --- | --- | --- | --- | --- | --- | --- | --- |
| vanRoessel; 2020 (Netherlands) ^64^ | Dutch Pancreatic Cancer Audit; 2014-2017 | 20 hospitals; (n = 3,341) | Pancreas; stage I-IV | Textbook outcome rate | Outcomes/  Adverse events | Compared to one another by funnel plot  Risk-adjusted: Yes | O:E ratio varied from 0.71 to 1.46 per hospital | No |
| Suraci; 2021  (USA) ^65^ | NCD; 2004-2016 | 401 hospitals; (n = 4,085) | Pancreatic head; stage I-II | RAMP | Technical | Compared to one another by O:E ratio  Risk-adjusted: Yes | Identified 12 low and 17 high O/E-ratio outlier facilities | No |
| Creighton; 2017 (Australia) ^66^ | New South Wales (NSW) Admitted Patient Data Collection & NSW Central Cancer Registry; 2005-2013 | 11 regions; (n = 6,537) | Pancreatic; stage I-IV | Resection rate  90-day mortality  5-year survival | Appropriateness  Outcomes/  Adverse events  Outcomes/  Adverse events | Compared to one another using mean as benchmark  Risk-adjusted: Yes | Proportion that underwent resection varied significantly between regions, ranging between 8% and 21% (P< 0.001) | No |
| Merkow; 2014 (USA) ^67^ | NCD; 2006-2009 | 1,002 hospitals; (n = 14,889) | Adeno-carcinoma; stage I-III | RAMP | Technical | Compared to one another by O:E ratio  Risk-adjusted: Yes | 21 (2.1 %) hospitals had lower-than expected and 17 (1.7 %) had higher-than-expected margin involvement. | No |
| Swords; 2019 (USA) ^68^ | NCD; 2004-2014 | 1,189 hospitals; (n = 63,640) | PDAC; stage I-II | Rate of curative-intent surgery | Appropriateness | Compared to one another using mean as benchmark  Risk-adjusted: Yes | Adjusted hospital rates of surgery varied 6-fold (11.4%–70.9%). | No |
| Nuckles; 2021 (USA) ^69^ | NCD; 2004-2015 | 852 hospitals; (n = 19,968) | Adeno-carcinoma; stage I-IV | RAMP | Technical | Compared to one another by O:E ratio  Risk-adjusted: Yes | 83 hospitals had higher than expected RAMP rates (poorer performing hospitals) and 53 had lower than expected RAMP rates (higher performing hospitals). | No |

*Upper Gastrointestinal Tract*

| **Study; Year (Country)** | **Data Source; period** | **Hospitals evaluated; (patients evaluated)** | **Cancer type(s); stage** | **Quality Indicator(s)** | **Domain** | **Categorization, identification of outliers** | **Key Findings** | **Feedback to studied groups?** |
| --- | --- | --- | --- | --- | --- | --- | --- | --- |
| In; 2015 (USA) ^70^ | NCD; 2007-2011 | 977 hospitals; (n = 15,796) | Esophageal; stage I-III | 30-day mortality  90-day mortality | Outcomes/  Adverse events  Outcomes/  Adverse events | Compared to one another by odds ratio  Risk-adjusted: Yes | Of the 96 hospitals that were ranked best for 30-day mortality, 46 hospitals changed their rank to ‘average and 1 hospital changed to worse when 90-day mortality was examined.  Of the 96 hospitals that were ranked as worst performing for 30-day mortality, 48 changed their rank to average when 90-day mortality was examined | Yes  Fed into QI efforts at national level through CQIP. |
| Merkow; 2012 (USA) ^71^ | NCD; 1998-2007 | 639 hospitals; (n = 13,995) | Esophageal; stage I-III | Adequate lymphadenectomy rate | Appropriateness | Compared to pre-determined benchmark  Risk-adjusted: Yes | At the hospital level, only 45 centers (7.0%) examined a median of at least 15 lymph nodes. | No |
| Voeten; 2020  (Netherlands) ^72^ | Dutch Upper GI Cancer Audit; 2011-2018 | 22 hospitals; (n = 5,894) | Esophageal; stage I-IV | Failure-to-cure rate | Outcomes/  Adverse events | Compared to one another via funnel plot  Risk-adjusted: Yes | 2 hospitals had statistically significant higher failure to cure percentages, whereas 2 had lower percentages | No |
| Portuondo; 2021 (USA) ^73^ | NCD; 2006-2015 | 987 hospitals; (n = 5,433) | Gastric; stage I-IV | Proportion of adequate lymphadenectomy (AL) = >15 nodes | Appropriateness | Compared to pre-determined benchmark  Risk-adjusted: Yes | 45.6% of hospitals met the AL performance benchmark. | No |
| Giambra; 2021 (USA) ^74^ | NCD; 2004-2016 | 1,245 hospitals; (n = 26,590) | Gastric; stage I-III | Proportion of operations w lymph node yield > 15 | Appropriateness | Compared to one another by O:E ratio  Risk-adjusted: Yes | 198 low outlier LN yield hospitals and 135 high outlier LN yield hospitals (observed-to-expected ratio of 0.42 0.24 versus 1.38 0.19, P < 0.0001). | No |
| Liu; 2018 (USA) ^75^ | NSQIP; 2010-2016 | 645 hospitals; (n = 204,732) | Upper GI tract; stage I-IV | 30-day complication rate | Outcomes/  Adverse events | Compared to one another by O:E ratio  Risk-adjusted: Yes | NCI‐CCs had comparable risk‐adjusted outcomes to non‐NCI‐CCs performing the same types of major cancer operations: the NCI‐CC and non‐NCI‐CC cohorts include both high‐ and low‐performers. | No |
| Liu; 2019 (USA) ^76^ | NSQIP & Hospital Consumer Assessment of Healthcare Providers and Systems (HCAHPS); 2015-2016 | 530 hospitals; (n = 60,526) | Upper GI tract; stage I-IV | 30-day death or serious morbidity (DSM) rate  Patient-reported experience (HCAHPS) | Outcomes/  Adverse events  Patient experience | Compared to one another by O:E ratio and HCAHPS score  Risk-adjusted: Yes | 38 highest- and 48 lowest performing hospitals along the two axes | No |

*Head & Neck*

| **Study; Year (Country)** | **Data Source; period** | **Hospitals evaluated; (patients evaluated)** | **Cancer type(s); stage** | **Quality Indicator(s)** | **Domain** | **Categorization, identification of outliers** | **Key Findings** | **Feedback to studied groups?** |
| --- | --- | --- | --- | --- | --- | --- | --- | --- |
| VandenBruel; 2013 (Belgium) ^77^ | Belgian Health Insurance database & Belgian Cancer Registry; 2004-2006 | 2 regions; (n = 1,913) | Thyroid; stage not reported | Rate of fine needle aspiration (FNA)  Proportion undergoing lymph node dissection synchronously | Appropriateness  Appropriateness | Compared regions on rate per 100,000 person years  Risk-adjusted: Yes | The rate of FNAC was similar, whereas the rate of surgery was lower in the low incidence region (34 [95% CI 33; 35 ] vs 80 [95% CI 79; 81 ] per 100 000 person years in the high incidence region; P < .05). | No |
| Schoppy; 2017 (USA) ^78^ | NCD; 2004-2013 | 1,008 hospitals; (n = 64,738) | Upper aerodigestive tract; stage I-IV | Rate of negative margins  Rate of adequate lymph node yield (18+) | Technical  Appropriateness | Compared to one another using mean as benchmark and pre-determined benchmark levels  Risk-adjusted: Yes | Most hospitals had high proportions of operations with negative margins (median, 76.1%; interquartile range [IQR], 66.7%-84.6%), but only 105 hospitals (10.4%) achieved a negative margin in 90% or more of cases.  199 hospitals (19.7%) achieved an 18+ lymph node count neck dissection in 80% or more of patients. | No |
| Wrenn; 2021 (USA) ^79^ | Collaborative Endocrine Surgery Quality Improvement Program (CESQIP); 2014-2019 | 43 hospitals; (n = 740) | Papillary thyroid ca;  Stage I-II | Proportion of tumors <4cm resected via total thyroidectomy | Appropriateness | Compared to one another using mean as benchmark  Risk-adjusted: No | Significant variation in thyroid lobectomy (vs total thyroidectomy) rate by institution via ANOVA (p < .001). | No |

*Prostate*

| **Study; Year (Country)** | **Data Source; period** | **Hospitals evaluated; (patients evaluated)** | **Cancer type(s); stage** | **Quality Indicator(s)** | **Domain** | **Categorization, identification of outliers** | **Key Findings** | **Feedback to studied groups?** |
| --- | --- | --- | --- | --- | --- | --- | --- | --- |
| Spencer; 2008 (USA) ^80^ | NCD & medical records; 2000-2001 | 9 regions; (n = 2,775) | Prostate; stage I-II | Facility employs at least 1 board certified urologist  Psychological counseling available  Pretherapy functional assessment completed  Treatment options and risks discussed  Adequate follow-up (2+ visits w/in 1yr treatment) | HC delivery  Access  Appropriateness  Patient experience  Appropriateness | Compared based on pre-determined benchmark  Risk-adjusted: Yes | Compliance rates were consistently high (>85%) for the structure indicators. Greater variation observed with process indicators, especially pretherapy evaluation indicators: assessment of pretreatment sexual (46.4%; 95% CI, 43.7% to 49.1%), urinary (78.4%; 95% CI, 76.3% to 80.5%), and bowel function (52.1%; 95% CI, 49.4% to 54.7%). | No |
| Abdollah; 2016  (USA) ^81^ | MUSIC; 2013-2014 | 42 hospitals; (n = 3,035) | Prostate; stage NA | Proportion of biopsies performed for men with limited life expectancy (LE) | Appropriateness | Compared to one another using mean as benchmark  Risk-adjusted: Yes | Proportion of biopsies performed for men with limited LE ranged from 3.8% to 39% across practices (p < 0.001) | Yes  Examining instituting life expectancy calculator to aid in treatment decisions. |
| Burks; 2017 (USA) ^82^ | MUSIC; 2012-2015 | 36 hospitals; (n = 5,375) | Prostate; stage NA | Incidence of repeat biopsy | Appropriateness | Compared to one another using mean as benchmark  Risk-adjusted: Yes | Incidence of repeat biopsy ranged from 0% to 17.2% across practices (p <0.001). | Yes  Fed into QI efforts at the regional level. |

*Multiple Tumor Types in One Study*

| **Study; Year (Country)** | **Data Source; period** | **Hospitals evaluated; (patients evaluated)** | **Cancer type(s); stage** | **Quality Indicator(s)** | **Domain** | **Categorization, identification of outliers** | **Key Findings** | **Feedback to studied groups?** |
| --- | --- | --- | --- | --- | --- | --- | --- | --- |
| Liu; 2020 (USA) ^83^ | NSQIP; 2013-2018 | 589 hospitals; (n = 6,203) | Any cancer type with peritoneal involvement; stage NA | Proportion experiencing DSM  30-day mortality rate  30-day serious morbidity rate  Unplanned reoperation rate  30-day unplanned readmission rate | Outcomes/  Adverse events  Outcomes/  Adverse events  Outcomes/  Adverse events  Outcomes/  Adverse events  Outcomes/  Adverse events | Compared to one another via odds ratio  Risk-adjusted: Yes | After adjusting for patient characteristics, hospital-level variation was detected between all hospitals and outcomes studied  Unadjusted rates of death, serious morbidity, reoperation, readmission, and DSM were 1.4%, 12.9%, 3.6%, 8.6%, and 13.4%, respectively. The corresponding coefficients of variation for hospital-level performance were 4.7%, 2.1%, 4.6%, 14.4%, and 1.0%. | No |
| Diaz; 2021 (USA) ^84^ | Billing data; 2014-2016 | 322 hospitals; (n = 57,458) | Colorectal, lung, pancreas; stage NA | Average total episode payment | Access | Compared to one another using mean as benchmark  Risk-adjusted: Yes | Average episode payments varied nearly as much within hospital systems for pancreatectomy ($1946 between the lowest and highest spending systems), lung resection ($625 between the lowest and highest spending systems), and colectomy ($813 between the lowest and highest spending systems) as they did between the lowest and highest spending hospitals (pancreatectomy: $2034; lung resection: $1789; and colectomy: $770). | No |
| Greenberg; 2011 (USA) ^85^ | SEER; 2000-2007 | 1,314 hospitals; (n = 75,965) | Breast, colon, gastric, rectal, or thyroid; stage I-IV | Proportion provided uniformly guideline-concordant care  Proportion receiving Total thyroidectomy for papillary cancer>1.5 cm or node positive  Proportion undergoing central neck dissection for node-positive papillary thyroid cancer  Proportion with gastric LN yield >15  Proportion with colon LN yield >12  Proportion undergoing axillary dissection for node-positive breast cancer | Appropriateness  Appropriateness  Appropriateness  Appropriateness  Appropriateness  Appropriateness | Compared to one another using pre-determined benchmark  Risk-adjusted: Yes | At least 50% of hospitals provided guideline-concordant care to 100% of their patients for 6 of 11 guidelines  Only 2 of 5 measures for nodal management had concordance rates greater than 90%. | No |
| Gore; 2012 (USA) ^86^ | Washington State Comprehensive Hospital Abstract Reporting System; 2003-2007 | 37 hospitals; (n = 853) | Prostate, kidney, bladder; stage I-IV | Occurrence of secondary infections, postoperative DVT/PE, wound dehiscence  Prolonged length of stay  90-day readmission rate  90-day mortality rate | Outcomes/  Adverse events  HC delivery &  Outcomes/  Adverse events  Outcomes/  Adverse events  Outcomes/  Adverse events | Compared to one another using odds ratios  Risk-adjusted: Yes | Hospital-level variation was most profound for LOS outcomes after nephrectomy and prostatectomy (variance in prolonged LOS, 8.1% and 26.7%, respectively), thromboembolic events after nephrectomy (8% of variance), and mortality after cystectomy (7.1% of variance)  For morbidity and mortality, there were no statistically significant outliers either positively or negatively in the outcomes analyzed after radical cystectomy  Several hospitals with higher nephrectomy volumes had length-of-stay outcomes that were worse than the state average. Readmission rates did not vary by hospital.  length-of-stay outcomes varied markedly after radical prostatectomy | No |
| Haneuse; 2018  (USA) ^87^ | California Cancer Registry; 2007-2011 | 351 hospitals; (n = 138,799) | Breast; Colorectal; Prostate; Head & Neck; lung, bladder, kidney, endometrial, pancreatic, liver, esophageal; stage I-III | In-hospital mortality  90-day mortality rate  90-day readmission rate | Outcomes/  Adverse events  Outcomes/  Adverse events  Outcomes/  Adverse events | Compared to one another using O:E ratios  Risk-adjusted: Yes | Across 260 hospitals with a mean annual surgical volume of 10 or more, 59 (22.7%) had lower-than expected rates for all 3 metrics, 105 (40.4%) had higher-than-expected rates for 2 of the 3, and 19 (7.3%) had higher-than-expected rates for all 3 metrics. | No |

*Other*

| **Study; Year (Country)** | **Data Source; period** | **Hospitals evaluated; (patients evaluated)** | **Cancer type(s); stage** | **Quality Indicator(s)** | **Domain** | **Categorization, identification of outliers** | **Key Findings** | **Feedback to studied groups?** |
| --- | --- | --- | --- | --- | --- | --- | --- | --- |
| Gynecologic | | | | | | | | |
| Gildea; 2016 (UK) ^88^ | Hospital Episodes and Statistics (HES); 2000-2009 | 148 hospitals; (n = 46,589) | Endometrial; stage I-IV | 30-day mortality | Outcomes/  Adverse events | Compared to one another via funnel plot  Risk-adjusted: Yes | Crude and casemix adjusted results found one hospital had a higher postoperative mortality rate than expected. | No |
| Polan; 2020 (USA) ^89^ | NCD; 2015 | 510 hospitals; (n = 20,670) | Endometrial; stage I-III | Proportion of resections done by minimally invasive surgery (MIS) | Technical | Compared to pre-determined benchmark  Risk-adjusted: Yes | In 283 (55%) hospitals ≥80% of hysterectomies were via MIS (overall MIS rate 89%). In the 227 hospitals that did not meet this metric, 61% of hysterectomies were performed using MIS. | No |
| Aletti; 2007 (USA) ^90^ | Medical records; 1994-2003 | 3 hospitals; (n = 564) | Ovarian; stage III-IV | 30-day major morbidity rate  Inability to receive planned chemotherapy  3mo mortality rate  LOS | Outcomes/  Adverse events  Outcomes/  Adverse events  Outcomes/  Adverse events  HC delivery &  Outcomes/  Adverse events | Compared to one another by O:E ratio  Risk-adjusted: Yes | After adjusting for relevant independent variables, 1 center appeared to have a O/E ratio statistically higher than 1 for 30-day major morbidity and LOS.  3mo mortality and inability to receive planned chemotherapy ratios were comparable. | No |
| Algera; 2023 (Netherlands) | Dutch Gynaecological Oncology Audit Registry; 2017-2020 | 22 hospitals (n = 1,865) | Ovarian; stage III-IV | Textbook outcome rate (Complete cytoreductive surgery, combined with the absence of 30-day mortality, severe complications, and prolonged length of admission (≥ten days)) | Outcomes/adverse events | Compared to one another by O:E ratio  Risk-adjusted: Yes | TO rates ranged from 40% to 69% between hospitals in the interval CRS cohort and 22% to 100% in the primary CRS cohort. In both analyses, one hospital had significantly lower TO rates (different hospitals). Case-mix adjustment significantly affected TO rates in the primary CRS analysis. | Yes  The DGOA is planning to facilitate round table discussions with all participating hospitals to investigate whether there are differences in care processes between the hospitals. Furthermore, the audit will assess whether interventions are needed to improve quality indicators, including textbook outcome. |
| Algera; 2023 (Netherlands) | Dutch Gynecological Oncology Audit | 21 hospitals (n = 1,822) | Ovarian; stage II-IV | Complicated course rate (any complication combined with a prolonged length  of hospital stay (>14 days), and/or complication requiring surgical, endoscopic, or radiological intervention, and/or any complication combined with prolonged intensive care unit stay (>1 day), and/or death within 30 days after the procedure, and/or death during hospital admission following surgery.) | Outcomes/adverse events | Compared to one another by O:E ratio  Risk-adjusted: Yes | Interhospital variation was not significant for case-mix factors. Complicated course rates ranged between 2.2% and 29.1%, and case-mix adjusted observed/expected ratios ranged from 0.20 to 2.67 between hospitals. Three hospitals performed outside the confidence intervals for complicated course rates. These hospitals remained outliers after case-mix adjustment. | Yes  Fed into efforts of Ductch Gyn Audit |
| Urologic | | | | | | | | |
| Lawson; 2017 (USA) ^91^ | NCD; 2004-2014 | 1,254 hospitals; (n = 131,319) | Renal cell; stage I-IV | Rate of MIS  Proportion of T1a tumors undergoing partial nephrectomy (PN)  RAMP for partial nephrectomy (PM)  LOS (radical nephrectomy)  30-day unplanned readmission rate (radical nephrectomy) (RP) | Technical  Appropriateness  Technical  HC delivery &  Outcomes/  Adverse events  Outcomes/  Adverse events | Compared to one another via funnel plot  Risk-adjusted: Yes | Statistically significant interhospital variation was observed for all QIs (p < 0.001) with between hospital variance proportions of 31%, 17%, 12%, 15%, and 20% for the MIS, PN, PM, LOS, and RP indicators, respectively. | No |
| AguilarPalacios; 2022 (USA) ^92^ | Admin data; 2005-2015 | 96 hospitals; (n = 8,896) | Renal; stage I-II | Proportion of cases for which 1) T1a tumors underwent partial nephrectomy and 2) T1-T2 tumors underwent minimally invasive radical nephrectomy | Appropriateness & Access | Compared to one another by O:E ratio  Risk-adjusted: Yes | Identified 25/33/38 hospitals with higher/lower/average performance, respectively | No |
| Cole; 2019 (USA) ^93^ | Nationwide Readmissions Database (NRD).; 2014 | 320 hospitals; (n = 6,157) | Bladder; stage I-IV | 30-day readmission rate | Outcomes/  Adverse events |  | The 30-d readmission rate was 29.5% (95% confidence interval [CI] 27.8–31.2%), ranging from 1.4% (95% CI 0.6–2.2%) in the bottom quartile to 73.6% (95% CI 68.4–78.7) in the top.  After adjusting for patient characteristics, hospital-level effects explained little of the large between-hospital variability in readmission rates | No |
| Dermatologic | | | | | | | | |
| Grange; 2008 (France) ^94^ | Regional clinical data registry & medical records; 2016 | 5 regions; (n = 710) | Melanoma; stage I-II | Rate of insufficient surgical margins  Average time from initial excision to definitive surgery  Proportion of patients with sentinel lymph node biopsy (SLNB) | Technical  Timeliness  Appropriateness | Compared to one another using mean as benchmark  Risk-adjusted: Yes | Certain geographic regions were outliers for excessive margins, time from excision to definitive surgery, and SLNB | No |
| vanEgmond; 2021 (Netherlands) ^95^ | National clinical data registry (Vektis); 2016 | 124 hospitals; (n = >220,000) | Skin cancer (all types); stage I-IV | Proportion treated via Mohs micrographic excision (MMS)  Average follow-up visits per patient | Appropriateness  Appropriateness | Compared to one another using mean as benchmark  Risk-adjusted: Yes | Practice variation of MMS was low under the 75th percentile, but outliers at the 100th percentile were detected, which indicates that few centers performed this far more often than average.  Number of follow-up visits did not vary much between the 25^th^-75^th^ percentile, but at the 100^th^ percentile it was significantly greater (6.61 vs 1.75) | No |

|  |  |  |
| --- | --- | --- |
|  | **No** | **(%)** |
| **Method of data presentation/visualization** |  |  |
| *Scatter chart with benchmark* | 28 | (31) |
| *Bar chart with benchmark* | 21 | (24) |
| *Funnel Plot* | 18 | (20) |
| *Bar chart without benchmark* | 5 | (6) |
| *Kaplan-Meier curve* | 5 | (6) |
| *Trendline* | 4 | (5) |
| *Heatmap* | 1 | (1) |
| *None* | 10 | (12) |

**Supplemental Table 6. Data visualization**
